# SARS-CoV-2 variants of concern: spike protein mutational analysis and epitope for broad neutralization

**DOI:** 10.1038/s41467-022-32262-8

**Published:** 2022-08-18

**Authors:** Dhiraj Mannar, James W. Saville, Zehua Sun, Xing Zhu, Michelle M. Marti, Shanti S. Srivastava, Alison M. Berezuk, Steven Zhou, Katharine S. Tuttle, Michele D. Sobolewski, Andrew Kim, Benjamin R. Treat, Priscila Mayrelle Da Silva Castanha, Jana L. Jacobs, Simon M. Barratt-Boyes, John W. Mellors, Dimiter S. Dimitrov, Wei Li, Sriram Subramaniam

**Affiliations:** 1 Department of Biochemistry and Molecular Biology, University of British Columbia, Vancouver, BC V6T 1Z3 Canada; 2 Center for Antibody Therapeutics, Division of Infectious Diseases, Department of Medicine, University of Pittsburgh School of Medicine, 3550 Terrace Street, Pittsburgh, PA 15261 USA; 3 Department of Infectious Diseases and Microbiology, Graduate School of Public Health, University of Pittsburgh, Pittsburgh, PA USA; 4 Division of Infectious Diseases, Department of Medicine, University of Pittsburgh School of Medicine, Pittsburgh, PA USA; 5 Gandeeva Therapeutics Inc., Vancouver, BC Canada

**Keywords:** Biochemistry, Structural biology

## Abstract

Mutations in the spike glycoproteins of SARS-CoV-2 variants of concern have independently been shown to enhance aspects of spike protein fitness. Here, we describe an antibody fragment (V_H_ ab6) that neutralizes all major variants including the recently emerged BA.1 and BA.2 Omicron subvariants, with a unique mode of binding revealed by cryo-EM studies. Further, we provide a comparative analysis of the mutational effects within previously emerged variant spikes and identify the structural role of mutations within the NTD and RBD in evading antibody neutralization. Our analysis shows that the highly mutated Gamma N-terminal domain exhibits considerable structural rearrangements, partially explaining its decreased neutralization by convalescent sera. Our results provide mechanistic insights into the structural, functional, and antigenic consequences of SARS-CoV-2 spike mutations and highlight a spike protein vulnerability that may be exploited to achieve broad protection against circulating variants.

## Abstract (web summary)

SARS-CoV-2 variants have accumulated multiple defining mutations within their spike glycoproteins. Here, the authors report a structural basis for broad neutralization of several variants by a heavy chain antibody fragment and provide a mutational analysis focusing on antibody evasion, receptor engagement, and spike protein structure.

## Introduction

Genomic surveillance of SARS-CoV-2 during the first year of the COVID-19 pandemic revealed that the D614G mutation in the spike glycoprotein (S protein) was the sole widespread consensus mutation, with the G614 genotype largely replacing the D614 genotype in February 2020^1,2^. In November 2020 however, the emergence of the Alpha (B.1.1.7) variant began capturing global headlines and coincided with a surge in COVID-19 cases in the United Kingdom. Within 4 months, the Alpha variant became the globally dominant SARS-CoV-2 lineage^1^. The emergence of the Alpha lineage was quickly followed by the emergence of the Beta (B.1.351), Gamma (P.1), and Epsilon (B.1.427/429) variants in early 2021, with the Kappa and Delta variants emerging shortly thereafter. The Delta variant achieved global dominance until it was replaced by the Omicron BA.1 sub-lineage in early 2022, which was swiftly replaced by the BA.2 sub-lineage of Omicron, followed by increasing prevalence of the BA.5 sub-lineage.

SARS-CoV-2 utilizes a trimeric spike glycoprotein for attachment to the host cell receptor angiotensin-converting enzyme 2 (ACE2) and for the subsequent cell entry step which involves the fusion of host cell and viral membranes. Given its crucial role in the viral replicative cycle, the spike protein represents an important therapeutic target and is a critical antigen in host immune responses. All emerging variants contain defining mutations within their spike proteins, with multiple mutations clustering within the receptor-binding domain (RBD) impacting both ACE2 binding and antibody neutralization escape^3–5^, while mutations within the highly antigenic loops in the N-terminal domain (NTD) across these variants reduce antibody neutralization^6^. Given the rapidly changing mutational and antigenic landscape of the SARS-CoV-2 spike protein, a structural understanding of spike protein mutational effects and the discovery of broadly neutralizing epitopes is of importance.

Here, we present an antibody fragment (ab6) with neutralization activity against multiple variants (Alpha, Beta, Gamma, Delta, Kappa, Epsilon, and Omicron) and report its epitope within the RBD using cryogenic electron microscopy (cryo-EM). This antibody epitope is remote from most VoC mutations, explaining its ability to confer pan-variant neutralization. Given the enhanced antibody escape of circulating variant spikes, the epitope we define here provides opportunities for rational therapeutic targeting of variant SARS-CoV-2 S proteins. We also report studies of spike structure, ACE2 affinity, and evasion of antibodies afforded by previously emerged variant spikes, providing a general structural rationale for enhanced viral fitness of the variants.

## Results

### Broad neutralization of the SARS-CoV-2 spike protein by an unconventional antibody fragment

**Fig. 1:**
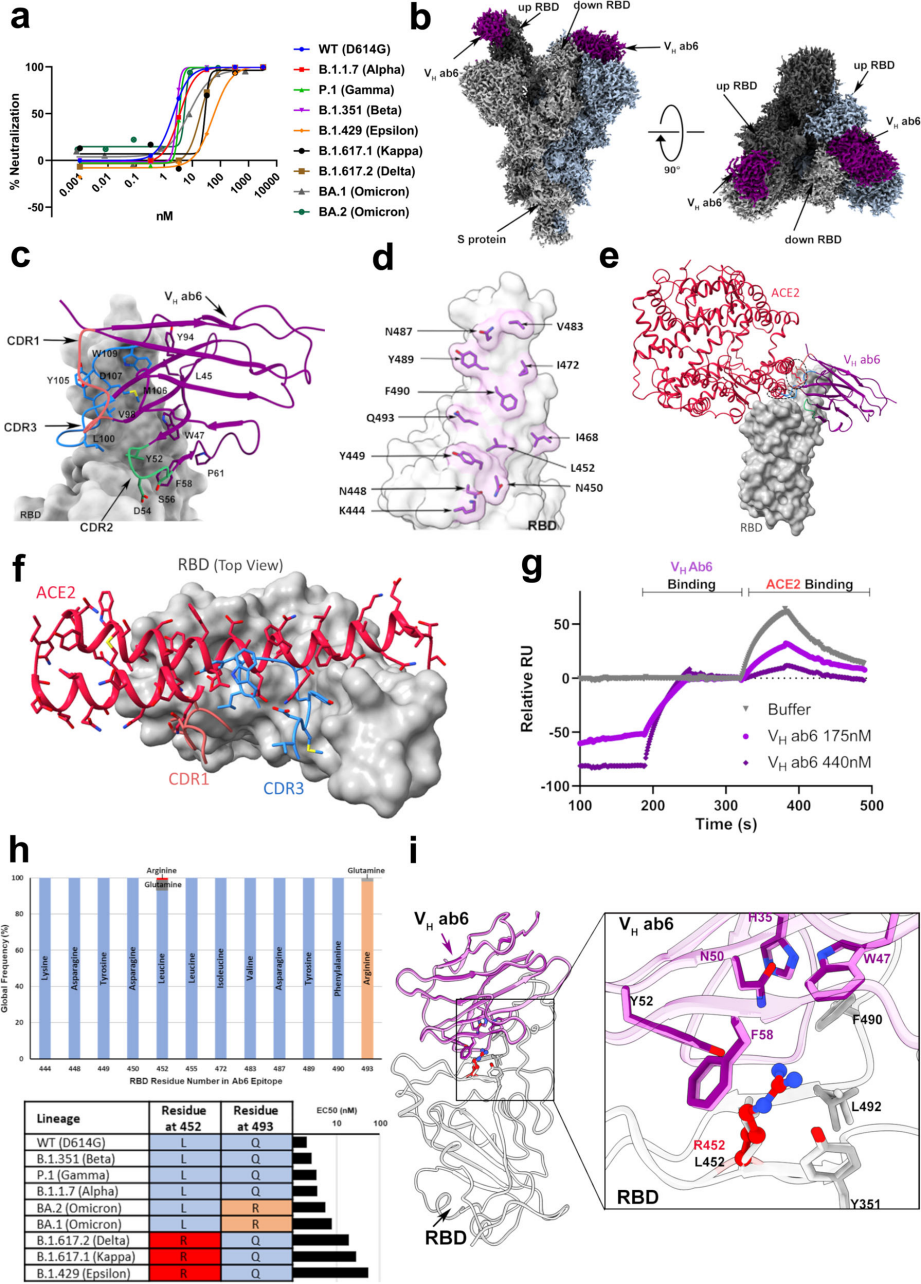
Ab6 broadly neutralizes SARS-CoV-2 variants via a largely conserved molecular epitope. **a** Pseudovirus neutralization of SARS-CoV-2 variants by V_H_ ab6, performed in at least technical triplicate (*n* = 3), the mean is plotted. **b** 2.4 Å global cryo-EM density map of V_H_ ab6 bound to wild-type S protein. Density corresponding to S protein protomers and ab6 are shown in grayscale and blue, respectively. **c** ab6 contact zones. The RBD and ab6 are shown as a gray surface and colorized cartoon, respectively. The ab6 scaffold is colored purple and complementarity determining regions (CDRs) of ab6 are colored as follows: CDR1—red; CDR2—green; CDR3—blue. **d** Footprint of ab6. The sidechains of footprint residues are shown in purple. **e** Overlap of ab6 and ACE2 binding footprints. The local refined model of the ab6-RBD interface was superposed with the crystal structure of the ACE2-RBD complex (PDB: 6M0J). ACE2 is shown in red while VH ab6 is shown as in **c**. The RBD is depicted as a gray surface. Models were aligned using the RBD. Ovals highlight steric clashing between ACE2 and V_H_ ab6. **f** Detailed view of clashes made by CDR3 and CDR1 of V_H_ ab6 with the N terminal helices of ACE2. **g** SPR-based spike protein competition assay between ACE2 and V_H_ ab6. Spike protein was loaded onto an SPR chip surface before buffer or indicated concentrations of V_H_ ab6 were injected, followed by injection of ACE2-FC. Relative response units (RUs) are plotted on the *Y* axis. **h** (Top) Global frequency of residue identity within the ab6 footprint in GISAID deposited sequences as of May 1st, 2022. (Bottom) Residue identity at positions 452 and 493 within SARS-CoV-2 variants and V_H_ ab6 half-maximal effective concentrations (EC50) from pseudoviral neutralization assays. **i** Focused view superpositions of the cryo-EM-derived atomic model of the Epsilon (B.1.429) and wild-type (D614G) S proteins bound to V_H_ ab6. Epsilon and wild-type RBDs are colored light and dark gray respectively, while purple and pink models refer to ab6-WT and ab6-Epsilon, respectively. The R452 mutation is highlighted in red. Source data are provided as a Source data file.

V_H_ ab6 is a phage-display-derived antibody with the unusual biochemical property of exhibiting enhanced RBD affinity as a monomeric fragment as compared to a bivalent fusion^7^ and was recently shown to exhibit tolerance to several circulating RBD mutations^8^. We first confirmed this anomalous property of ab6, showing that the bivalent V_H_-Fc fusion has lower neutralization potency relative to the monovalent V_H_ construct in both pseudotyped and live virus neutralization assays (Supplementary Fig. 1a–c). We next assessed V_H_ ab6 binding and neutralization of variant spikes, (Fig. 1a and Supplementary Fig. 1d). Ab6 neutralized all variant spike pseudotyped viruses but exhibited 9–26-fold decreased potency for Epsilon, Kappa, and Delta and 4- and 3-fold lower potency for the BA.1 and BA.2 Omicron spikes respectively. We additionally assessed ab6 neutralization of Alpha, Beta, and Delta live viruses via authentic virus neutralization assays, confirming the lower potency of ab6 for the Delta variant (Supplementary Fig. 1b, c).

We next determined the cryo-EM structure of V_H_ ab6 bound to the WT spike at 2.57 Å (Supplementary Fig. 2), showing that ab6 binds to the RBD in both the up and down positions, via a unique binding mode (Fig. 1b). Local refinement of the down RBD bound by V_H_ ab6 enabled visualization of the ab6-RBD interface at 3.21 Å (Supplementary Fig. 2g, h) and revealed that the ab6-RBD interaction is dominated by contacts with the ab6 beta-sheet scaffold, which wraps around the RBD, extending this large interface to include its CDR2 and CDR3 loops but leaving the CDR1 loop free (Fig. 1c). This scaffold-mediated interaction necessitates a near perpendicular angle of approach for ab6 relative to the RBD, which likely can only be accommodated by a single V_H_ within a bivalent fusion construct. Furthermore, accessibility to the V_H_ scaffold may be limited within a bivalent fusion construct. Thus, the unusual angle of approach and dominance of scaffold-mediated contacts may account for the lower potency of the V_H_-Fc ab6 construct relative to V_H_ ab6.

The ab6 footprint involves multiple RBD residues (Fig. 1d) and overlaps that of ACE2, consistent with a mechanism of neutralization via ACE2 competition^7^ (Fig. 1e). The CDR1 and CDR3 loops of ab6 occupy positions that result in clashes with ACE2 upon superposition with an ACE2-bound RBD, with the CDR3 region directly competing with the amino terminal helix of ACE2 for RBD binding contacts, while the CDR1 loop poses a steric clash with the second helix of ACE2 without making RBD contacts (Fig. 1f). To confirm the ACE2 competitive nature of ab6 we employed competition ELISA (Supplementary Fig. 3) and competitive SPR experiments (Fig. 1g). Both experiments demonstrate the ability of ab6 to compete with ACE2 for spike protein binding.

Analysis of the ab6 footprint reveals the inclusion of L452 and Q493, consistent with the reduced potencies against the Epsilon, Delta, and Kappa spikes, which harbor the L452R mutation, along with the BA.1 and BA.2 Omicron sub-lineages which harbor the Q493R mutation (Fig. 1d, h). Genomic sequences from the GISAID database confirms the conserved nature of the ab6 epitope, highlighting the Q493R mutation to be the only significantly occurring variation in circulating variants as of May 1st, 2022. (Fig. 1h). Analysis of the relative neutralization potencies of L452R and Q493R containing variants suggests that ab6 exhibits greater sensitivity to the L452R mutation (Fig. 1h). To uncover the structural basis for the attenuation of ab6 potency by the L452R mutation, we obtained the cryo-EM structure of the Epsilon spike bound to ab6. A global 3D reconstruction was obtained at 2.45 Å (Supplementary Fig. 4), in which ab6 bound both up and down RBDs, as seen in the WT-ab6 complex. Focused refinement enabled visualization of the ab6-Epsilon spike interface at 3 Å (Supplementary Fig. 4g, h), revealing R452 to extend towards the ab6 scaffold (Fig. 1i and Supplementary Fig. 4i). This orientation places the positively charged R452 sidechain in close proximity to a hydrophobic portion of ab6, centered around F58. Thus, the reduced potency observed for R452-containing spikes is likely a result of unfavorable charge and steric effects. The Q493R mutation places R493 in close proximity to the ab6 CDR3 loop, and accommodation of this mutation may involve similar charge and steric penalties which give rise to the attenuation in ab6 potency against the BA.1 and BA.2 variant spike proteins.

To contextualize the broad-spectrum activity of ab6 we sought to investigate variant spike mutational effects on antibody evasion, receptor engagement, and spike structure. As we have previously reported these analyses on the Kappa and Delta variant spikes^9^, along with the BA.1 Omicron spike^10^ we proceed here with our analysis of the Alpha, Beta, Gamma, and Epsilon variant spike proteins.

### RBD- and NTD-directed antibodies are escaped by variant spike proteins

**Fig. 2:**
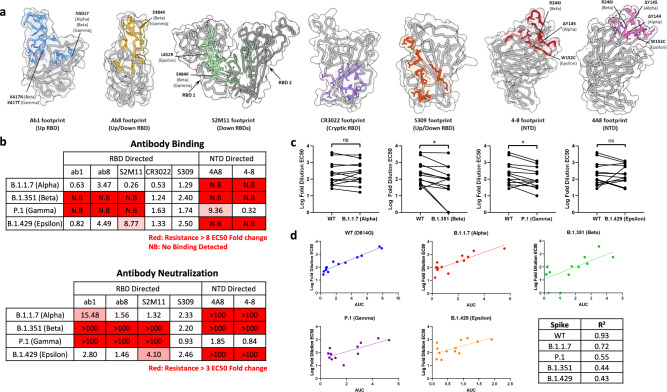
Alpha, Beta, Gamma, and Epsilon S proteins exhibit differences in monoclonal and polyclonal antibody escape. **a** Antibody-binding footprints for monoclonal antibodies included in the study. Variant S protein mutations falling within each footprint are highlighted. **b** Fold-changes in antibody binding (top) and pseudovirus neutralization EC50s (bottom) for each variant spike relative to wild-type (D614G). Antibody binding was quantified by ELISA. **c** Log-fold EC50 dilutions of patient sera when neutralizing wild-type and variant spike pseudovirus. Statistical significance was tested via the Wilcoxon matched pairs test (*p ≤ 0.05, ns, not significant) The *p* values for WT vs Alpha, WT vs Beta, WT vs Gamma, and WT vs Epsilon comparisons are 0.946, 0.0081, 0.0081, and 0.6848, respectively. See Supplementary Fig. 8 for patient information. **d** Correlation between NTD + RBD binding antibody levels and neutralization of wild-type and variant pseudoviruses. (AUC = area under the curve of the NTD + RBD ELISA binding curves). Correlation coefficients (*R*^2^) are tabulated in the bottom right of **d**. Source data are provided as a Source data file.

Having demonstrated the broad neutralization of variant spikes by ab6, we next aimed to provide a comparative analysis of spike mutational effects and antibody breadth using a representative panel of previously reported monoclonal antibodies. We selected RBD-directed antibodies^11–14^ which cover the four distinct anti-RBD antibody classes^15^ and an ultrapotent antibody, S2M11^16^, which uniquely binds two neighboring RBDs simultaneously (Fig. 2a). We additionally included the NTD-directed antibodies 4–8 and 4A8 to investigate the impact of NTD mutations within these variant spikes. (Fig. 2a). Antibody binding was quantified via enzyme-linked immunosorbent assay (ELISA), and compared with neutralization, which was measured via a pseudoviral entry assay (Fig. 2b). S309 and CR3022 are cross-reactive SARS-CoV-1 directed antibodies whose footprints do not span VoC mutations, and accordingly exhibited relatively unchanged binding across all variant spikes. We have previously characterized the mutational sensitivity of ab1, ab8, and S2M11 to spikes bearing only RBD mutations, and the current analysis of antibody evasion using spikes bearing all VoC mutations is consistent with our previous report^3^: (1) The N501Y mutation within the Alpha variant reduces but does not abolish the potency of ab1, while dramatic loss of ab1 activity is seen in Beta and Gamma variants due to mutation of K417 to N or T, respectively; (2) The E484K mutation abrogates ab8 activity in the Beta and Gamma variants; and (3) the L452R mutation reduces but does not abrogate activity of S2M11 in the Epsilon variant spike, drawing similarity to the mutational sensitivity of ab6. To compare the structural basis for the effect of L452R on S2M11 and ab6, we performed cryo-EM studies on the Epsilon-S2M11 complex (Supplementary Fig. 6). We obtained a global 3D reconstruction of the Epsilon spike bound to three S2M11 Fabs at 2.16 Å. In contrast to the Epsilon-ab6 structure wherein R452 protrudes into the antibody interface (Fig. 1i), R452 extends away from the S2M11 interface (Supplementary Fig. 7). As the L452 sidechain is accommodated within the footprint of S2M11, this positioning of R452 is likely due to steric clashing and charge repulsion effects which may increase the interaction energy and underlie the observed attenuation of potency.

Evasion of NTD-directed antibodies was observed in cases when mutations were either within, or adjacent to, antibody footprints (Fig. 2b), corroborating the recently described remodeling of antigenic loops in the Alpha, Beta, and Epsilon spikes^17,18^. The W152C substitution within the Epsilon NTD is inside the footprints of 4A8 and 4–8, and both antibodies escaped by this variant spike. The Beta NTD contains a deletion (Δ242–245) which spans the 4–8 footprint, along with the R261I substitution spanning both 4A8 and 4–8 footprints, leading to escape from both antibodies. The footprint of 4A8 and 4–8 spans a deleted site within the Alpha NTD (Δ144–145) leading to escape. These direct and allosteric mutational effects are consistent with previous findings on NTD rearrangement within these variants and demonstrate their antibody evasive properties.

Having characterized monoclonal antibody evasion by variant spikes, we extended our analysis to include polyclonal antibody escape from human sera. Sera were collected from a spectrum of patients with varying COVID-19 infection histories and vaccination statuses (Supplementary Fig. 8a) and subjected to neutralization and binding assays (Supplementary Figs. 8b, c and 9). Potent neutralization of WT spike pseudovirus was observed in all COVID-19 positive or vaccinated samples but not with pre-pandemic sera from uninfected patients, suggesting limited pre-existing immunity (Supplementary Fig. 8b, c). While serum levels of spike ectodomain binding antibodies correlated poorly with wild-type spike neutralization, strong correlations were observed between NTD and RBD binding antibody levels and neutralization (Supplementary Fig. 10), corroborating the dominance of neutralizing epitopes within the NTD and RBD^15,19,20^. We observed various effects on neutralization escape when sera samples were assayed using variant spike pseudotyped viruses, obtaining statistically significant decreases in neutralization efficacy for both Beta and Gamma variants relative to WT (Fig. 2c). Interestingly, the high correlation between serum NTD and RBD binding antibodies and pseudovirus neutralization for wild-type spikes was markedly reduced for all variant spikes (Fig. 2d). Taken together, these results highlight the role of mutations within the NTD and RBD of variant spikes in driving evasion of SARS-CoV-2 directed monoclonal and polyclonal antibodies, providing a basis to evaluate the broad mutational tolerance exhibited by S309 and ab6.

### Enhanced receptor binding by variant spike proteins

In addition to driving antibody escape, variant spike mutations can enhance receptor engagement, which may underlie increases in infectivity. To investigate the ACE2 binding potential of SARS-CoV-2 variant spikes, recombinant S protein ectodomains bearing variant spike mutations were used in biolayer interferometry (BLI) experiments. All mutant spikes exhibited slightly higher affinities for immobilized dimeric Fc-ACE2 when compared to wild-type (Supplementary Fig. 11a, b). Additionally, we used flow cytometry to evaluate the ability of recombinant dimeric Fc-ACE2 to bind wild-type or variant full-length spikes which we transiently expressed in Expi-293 cells. We did not observe major differences in spike protein expression across the variants (Supplementary Fig. 12a), and all mutant spikes tested demonstrated marginally enhanced ACE-2 binding potencies relative to wild-type (Supplementary Fig. 11c). These complementary assays demonstrate that the totality of mutations within each variant S protein enable slightly enhanced ACE2 binding, suggesting a contributing factor for the increased infectivity observed for these SARS-CoV-2 variants.

### Structural effects of variant spike protein mutations

**Fig. 3:**
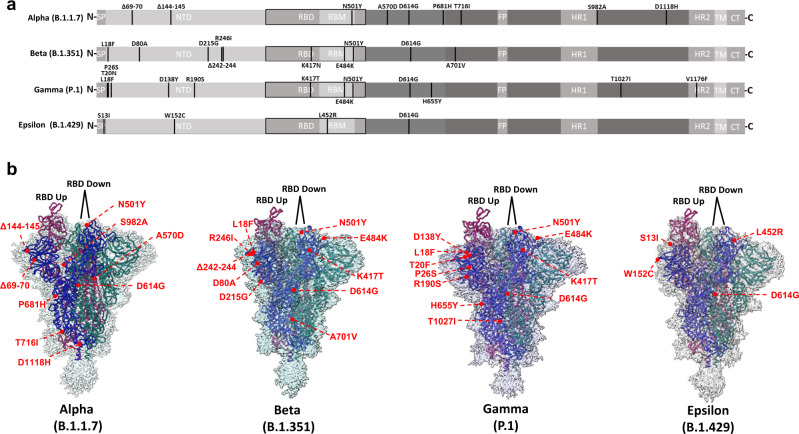
Cryo-EM structures of Alpha, Beta, Gamma, and Epsilon spike glycoproteins. **a** Linear schematic depicting mutations within variant S proteins (SP signal peptide, NTD N-terminal domain, RBD receptor binding domain, RBM receptor binding motif, FP fusion peptide, HR1 Heptad repeat 1, HR2 Heptad repeat 2, TM transmembrane, CT cytoplasmic tail). **b** Global cryo-EM maps and models for the Alpha (2.56 Å), Beta (2.56 Å), Gamma (2.25 Å), and Epsilon (2.4 Å) variant S proteins. Mutational positions are indicated and labeled in red.

Having demonstrated mutational effects on antibody evasion and receptor engagement, we next sought to characterize the structural impacts of variant S protein mutations. To this aim, ectodomains bearing variant spike mutations were used for cryo-EM structural studies. Global 3D reconstructions were obtained at resolutions ranging from (2.25–2.5 Å) (Supplementary Figs. 13–16), yielding open trimers with one RBD in the up conformation and 2 RBDs in the down conformation for all spikes (Fig. 3). The resolution within the NTD and RBD was insufficient for accurate visualization of mutational impacts within these domains, due to high degrees of conformational heterogeneity. In contrast, we were able to confidently model sidechains close to, and within the S2 domain, owing to its limited flexibility. We therefore first focused our analysis on mutational effects within this region, which predominantly localized to inter-protomer interfaces.

**Fig. 4:**
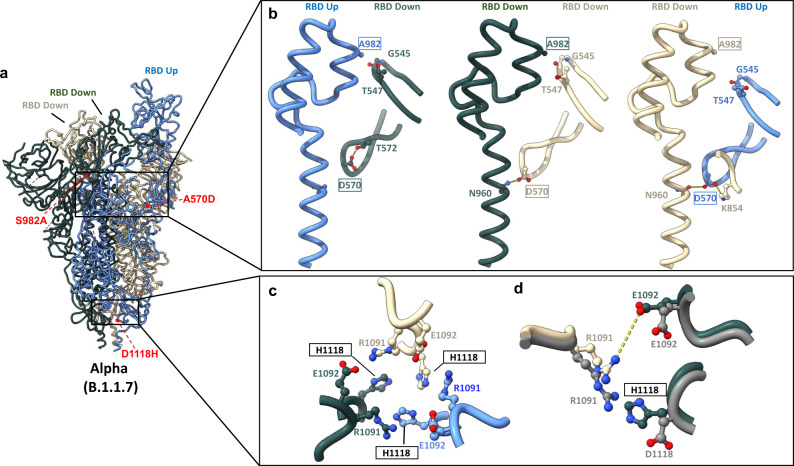
Alpha variant S protein mutations result in structural changes at inter-protomer interfaces. **a** Global model of the Alpha variant S protein with one RBD in the “up” conformation (blue) and two RBDs in the “down” conformation (beige and gray). Relevant mutations at inter-protomer interfaces are highlighted in red. **b** Modulation of inter-protomer contacts by mutations S982→A and A570→D between protomers with differential RBD conformations. **c**, **d** Impact of the D1118→H mutation on inter-protomer contacts. **c** model of H1118 and adjacent residues within the Alpha spike. **d** Superposition demonstrating differences between the Alpha spike (colored) and the wild-type spike (PDB: 6VSB) (shown in gray). Potential interactions (either electrostatic or hydrogen bonding) are highlighted as dashed lines. Mutated residues are indicated as boxed labels.

Inspection of the structure of the Alpha variant shows that the A570D and S982A mutations appear to contribute protomer-specific structural effects with implications for RBD positioning (Fig. 4b). Within both “RBD down” protomers, D570 either occupies a position within hydrogen bonding distance of N960 within the adjacent “RBD down” protomer or sits within intra-protomer hydrogen bonding distance with T572 when the RBD of the adjacent protomer is in the up conformation (“RBD up”). D570 within the “RBD up” protomer uniquely forms a salt bridge with K854 in the adjacent “down RBD” protomer. S982 sits within the hydrogen bonding distance of residues G545 and T547 within adjacent “RBD down” protomers only, and such interactions are not possible with the S982A mutation. Thus, possibilities for the effects of the A570D and S982A mutations include allosteric modulation of RBD conformation through (1) addition of the K852–D570 salt bridge, and (2) loss of stabilizing interactions afforded by S982, in agreement with recent reports^4,21,22^.

The D1118H mutation within the Alpha variant enables local side chain rearrangements, giving rise to additional interprotomer contacts, via pi-cation interactions between R1091 and H1118 of adjacent protomers, and electrostatic interactions between R1091 and adjacent E1092 residues (Fig. 4c). Superposition of wild-type and Alpha spike models clearly demonstrates the differential positioning of H1118 compared to D1118, and the resulting movement of adjacent R1091 towards E1092 (Fig. 4d). These additional interprotomer contacts enabled by the D1118H mutation may aid in the stabilization of the Alpha spike protein in its trimeric form.

Additional mutations were visualized within the Beta and Gamma variant spike proteins, with implications for inter-protomer and intra-protomer contacts. The A701V mutation in the Beta variant S protein lies at the protein’s surface and the larger sidechain conferred by V701 may enable tighter interprotomer packing with Q787 on adjacent protomers (Supplementary Fig. 17a). The T1027I mutation lies within the Gamma spike S2 core and the bulky hydrophobic I1027 sidechain faces inwards, increasing the hydrophobicity of the local buried environment (Supplementary Fig. 17b). This may decrease the local dielectric constant, enhancing the strength of the nearby network of interprotomer salt bridges between E1031 and R1039 on adjacent protomers (Supplementary Fig. 17b) and thus stabilize the Gamma variant trimer.

To identify structural mutational impacts on ACE2 binding we next determined the cryo-EM structures of variant spike-ACE2 complexes. The resulting maps were obtained at average resolutions of ~2.6–3 Å (Supplementary Figs. 18–21). Focus-refined structures of the ACE2-RBD interface enabled visualization of RBD mutations, revealing local structures which are identical to our previously reported structures using spikes harboring variant RBD mutations alone^3^. Superposition of local RBD-ACE2 complex models revealed no significant structural changes (Supplementary Fig. 22). These structural findings confirm that mutations outside of the RBD do not modulate the positioning of ACE2-contacting residues at the receptor interface via allosteric mechanisms.

### Structure of the gamma variant NTD

**Fig. 5:**
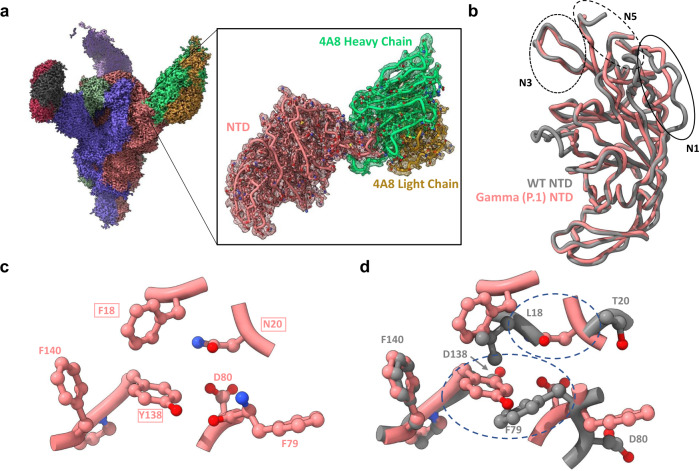
Structure of the Gamma variant NTD reveals rearrangement of the N1 loop. **a** Global cryo-EM density map of the Gamma variant S protein bound to 4A8 at 2.59 Å (left), and a focus-refined map and model for the Gamma NTD-4A8 complex at 2.66 Å (right). **b** Superposition of 4A8-bound Gamma and wild-type NTD models showing N1 loop rearrangement. The three loops (N1, N3, N5) comprising the “NTD neutralization supersite” are indicated with circles. **c** Positioning of the L18→F, D138→Y, and T20→N mutations and adjacent residues in the Gamma NTD. **d** Superposition of residues shown in **c** with WT residues demonstrates steric incompatibilities. Areas of steric clashes are indicated by dashed ovals. Mutated residues are indicated as boxed labels. The wild-type—4A8 model (PDB: 7C2L) was used for all superpositions and is shown in gray throughout the figure.

The structure of NTD region in the Alpha, Beta, and Epsilon variants has been previously reported^17,18^. Here, we report structural analysis of the NTD region in the Gamma variant, stabilized using Fab fragments of the NTD-directed antibodies 4A8^23^ and 4–8^24^. We obtained Cryo-EM reconstructions for Gamma spike protein–Fab complexes with all 3 NTDs bound for both 4A8 (Fig. 5a) and 4–8 (Supplementary Figs. 23 and 24). The bound antibody fragments improve the resolution of this flexible domain upon focused refinement, enabling the determination of structures at resolutions of ~2.6 Å both for the overall spike and the NTD-antibody interface (Supplementary Figs. 23 and 24).

Superposition of 4A8-bound wild-type and Gamma NTDs reveals remodeling of the antigenic supersite N1 loop^25^ within the Gamma variant but not the N3 and N5 loops, which comprise the majority of the 4A8 binding site (Fig. 5b). Analysis of nearby mutational effects provides additional reasoning for conformational remodeling of the N1 loop. The mutations L18F, D138Y, and T20N cluster close together, forming multiple interactions which stabilize the alternate N1 conformation (Fig. 5c). Namely, F18 and Y138 form an interconnected network of T-shaped pi stacking interactions with each other and the adjacent F140 residue. Additionally, Y138 and N20 sit within hydrogen bonding distance of the main chain carbonyl of F79 and the sidechain of D80, respectively. Comparison of sidechain positioning between wild-type and gamma structures in this region reveals steric clashes between Gamma residue Y138 and wild-type residues F79 and L18, and between Gamma residue N20 and the main chain of wild-type residue L18, resulting in differential positioning of D80 and F79 in the Gamma NTD (Fig. 5d). Identical positioning of the N1 loop is observed in the Gamma NTD-4-8 structure, further confirming these mutational effects (Supplementary Fig. 25). Thus, the unique interactions conferred by mutations within the Gamma NTD stabilize local conformations which are sterically incompatible with wild-type N1 positioning, causing N1 loop rearrangement.

## Discussion

Mutational enhancement of SARS-CoV-2 viral fitness can arise from effects on receptor engagement and evasion of neutralizing antibodies, with structural origins in the spike glycoprotein. Here we have examined these effects, demonstrating domain-specific differences in the roles and structural mechanisms of S protein mutations. Although such mutational changes can pose threats to natural and vaccine-induced immunity, the existence of preserved epitopes within functional domains holds great potential for future antigenic focus. This is highlighted in our analysis of variant SARS-CoV-2 spikes, which despite exhibiting effects on antibody evasion and ACE2 binding, shared a conserved epitope within the RBD which conferred broad neutralization.

The structural impacts of VoC S protein mutations offer insight regarding the differing mutational heterogeneity observed for the NTD and RBD. While VoC mutations within the RBD are limited to only substitutions, the NTD hosts a large array of deletions and substitutions, along with one documented insertion as seen in the BA.1 subvariant. These NTD mutations predominantly localize to the three loops constituting the “NTD neutralization supersite” (N1: residues 14–26, N3: residues 141–156, N5: residues 246–260)^25^. Our structures of VoC S proteins in complex with ACE2 demonstrate minimal structural changes in the RBD, reflecting its functional constraints in cell attachment, only permitting mutations that preserve the ACE2 binding interface. In contrast, our structure of the Gamma NTD confirms the role of mutations within this domain as enabling structural rearrangement of antigenic loops, a feature common to all variant spike NTDs (Alpha, Beta, Delta, Epsilon, BA.1, BA.2)^17,18,26–29^. These rearrangements are likely directed primarily by immune-evasive pressures. Taken together, these contrasting structural effects between variant NTD and RBD mutations likely arise due to different functional requirements and selective pressures between these domains.

Despite these domain-specific mutational pressures, several lines of evidence have emerged from the present study demonstrating the existence of pan-variant epitopes. The high correlation between RBD + NTD binding antibody levels and viral neutralization potency reflects the dominance of neutralizing epitopes within these domains of the WT spike (Fig. 2d). The diminished correlation between these parameters when assessing variant spikes demonstrates mutational escape within these domains. However, the fact that neutralization of variant spikes is attenuated, but not abolished, suggests the preservation of neutralizing epitopes. The existence of such epitopes within the RBD is corroborated by the unaltered potency of the SARS-CoV-1 directed antibody S309 across Alpha, Beta, Gamma, and Epsilon spikes. Most importantly, we reveal an epitope within the RBD conferring broad neutralization of all variants of concern spike proteins, including the rapidly spreading BA.2 subvariant. This epitope has largely survived viral evolution thus far, with the only prominent mutational changes being L452R and Q493R. These mutations are accommodated by ab6, albeit yielding decreased neutralization potencies. Therefore, we highlight this epitope for focus in the design of broadly protecting therapeutic antibodies and immunogens.

A comparison of ab6 to several other reported RBD-directed V_H_ domains highlights the unique epitope and mechanism of binding exhibited by ab6 (Supplementary Fig. 26). While VH fragments ab8^14^, H3^30^, and C5^30^ approach the RBD with more acute angles relative to ab6, C1^30^, and n3113^31^ both exhibit near perpendicular angles of approach involving some scaffold interactions, similar to ab6. Although the H3 footprint overlaps significantly with that of ab6, it is completely escaped by mutations within the Beta variant spike protein, unlike ab6. C1 binds an epitope distal to that of ab6 and the ACE2 binding site, yet is able to compete for ACE2 binding due to steric effects, whereas n3113 binds an epitope which overlaps with that of ab6 but is noncompetitive with regards to ACE2 binding. The neutralization breadth of n3113 extends to multiple variants^31^, consistent with its epitope overlap to that of ab6. Of note, n3113 was shown to bind exclusively to the RBD in the up conformation^31^ in contrast to ab6 which can recognize both down and up RBD conformations. Thus, distinguishing features of ab6 include its ability to adopt a near perpendicular angle of approach relative to the RBD in both up and down conformations via scaffold-mediated interactions, while also utilizing its CDR regions to compete with ACE2 via steric effects (CDR1) and direct binding of ACE2 interacting residues on the RBD (CDR3).

Analysis of the ab6 sequence using IMGT^32^ indicates the most closely aligning VH gene to be VH3-30 (Supplementary Fig. 27A). Comparison of the ab6-RBD structure to that of several other VH3-30 encoded RBD-directed antibodies demonstrates heterogeneity in the RBD epitopes recognized by VH3-30 encoded fragments (Supplementary Fig. 27B). Of these antibodies, the footprint of the heavy chain of antibody P17 shares the highest extent of overlap with that of ab6. In contrast to ab6, P17 potency was shown to be sensitive to mutations at position 484 within the RBD and was significantly escaped by the BA.1 Omicron subvariant^33^.

**Fig. 6:**
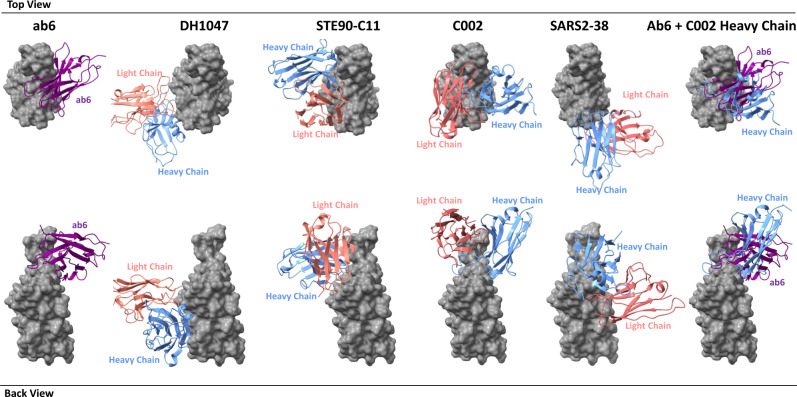
Footprint comparison between ab6 and selected RBD-directed antibodies. The RBD is depicted as a gray molecular surface and antibodies are depicted as colorized cartoon models. The following PDB files were utilized: 7LD1 (DH1047), 7B3O (STE90-C11), 7K8T (C002), 7MKM (SARS2-38). The RBD model from the ab6-RBD complex is shown for all antibody complexes for ease of visualization. For superpositions, structures were aligned using the RBD.

Several RBD mutation-resistant antibodies against SARS-CoV-2 have been reported during the preparation of this manuscript^34–38^, providing additional context regarding the conserved epitope we report here. Antibodies DH1047^39^ and STE90-C11^36^ were isolated from convalescent patients and SARS2-38^34^ from immunized mice. All three antibodies are RBD directed and bind epitopes distal to that of ab6 (Fig. 6). While STE90-C11 tolerated most circulating RBD mutations, it exhibited loss of activity against the K417T, K417N, and N501Y mutations^36^, which are present in many VOC/VOI spike proteins. In contrast, SARS2-38 and DH1047 bind highly conserved epitopes, retaining potency across all VOC/VOI spikes, with DH1407 exhibiting cross-reactivity with additional sarbecoviruses^34,38^. V_H_ ab6 is distinguished from these previously reported antibodies by its unique angle of approach and binding mode involving multiple V_H_ scaffold - RBD contacts (Fig. 1e), along with its small (15 kDa) size. Small antibody fragments are attractive therapeutic modalities given their enhanced tissue penetration compared to conventional monoclonal antibodies^40,41^.

A recent study by a global consortium defined seven RBD binding antibody communities and showed broadly neutralizing antibodies either bind cryptic epitopes within the inner RBD face (communities RBD-6, RBD-7), or are non-ACE2 competing antibodies that bind the outer RBD face (community RBD-5)^37^. Ab6 binds the inner RBD face and contacts the RBM, enabling ACE2 competition, drawing similarity to the RBD-4 antibody community, which interestingly was not shown to contain any broadly neutralizing antibodies. Structural comparison of the ab6 footprint with a representative RBD-4 antibody (C002)^15^ reveals an overlapping footprint shared by the C002 heavy chain and ab6 despite differences in binding modes (Fig. 6). C002 is derived from a convalescent patient, suggesting the potential for such an epitope to be recognized by natural antibodies. This evidence further supports the potential value of focus on the ab6 binding epitope for future therapeutic design.

## Methods

### Plasmids and cloning

SARS-CoV-2 S protein Alpha, Beta, Gamma, and Epsilon genes were synthesized and inserted into pcDNA3.1 (GeneArt Gene Synthesis, Thermo Fisher Scientific). Alpha, Beta, Gamma, Epsilon, Delta, and Kappa mutated S protein ectodomain genes were synthesized incorporating the hexa-proline stabilizing mutations, the transmembrane domain replaced with a fibrin trimerization motif, and a C-terminal hexa-histidine tag (GeneArt Gene Synthesis, Thermo Fisher Scientific). The HexaPro expression plasmid was a gift from Jason McLellan (Addgene plasmid #154754; http://n2t.net/addgene:154754; RRID: Addgene_154754) which we used to construct the D614G mutant via site-directed mutagenesis (Q5 Site-Directed Mutagenesis Kit, New England Biolabs).

C-terminal 7x his tagged NTD (amino acids 1–305) and RBD (amino acids 319–541) constructs were PCR amplified from D614G, Alpha, Beta, Gamma, and Epsilon full-length spike ORFs. NTD constructs were cloned into pcDNA 3.1 using NheI and MssI restriction enzyme cloning, while the RBD constructs were introduced in frame to the mu phosphatase signal sequence and incorporated within pcDNA3.1 via Gibson assembly (NEBuilder HiFi DNA Assembly Cloning Kit, New England Biolabs).

Human ACE2 (residues 1–615) with a C terminal 7x his tag was amplified from “hACE2”, a kind gift from Hyeryun Choe (Addgene plasmid # 1786) and cloned into pcDNA3.1 via BstXI and XbaI restriction enzyme cloning. Successful cloning was confirmed by Sanger sequencing (Genewiz, Inc.) for all constructs.

### Ethics oversight

Patient-derived sera samples were collected according to the CARE COVID Study (http://www.bccdc.ca/health-professionals/clinical-resources/covid-19-care/covid-19-serology-care-covid-study) with ethics approval from the UBC Clinical Research Ethics Board. Informed consent was received from all study participants.

### Pseudovirus neutralization assay

Variant and D614G spike pseudotyped retroviral particles were produced in HEK293T cells as described previously^42^. Briefly, a third-generation lentiviral packaging system was utilized in combination with plasmids encoding the full-length SARS-CoV-2 spike, along with a transfer plasmid encoding luciferase and GFP as a dual reporter gene. Pseudoviruses were harvested 60 h after transfection, filtered with a 0.45 µm PES filter, and frozen. For neutralization assays, HEK293T-ACE2-TMPRSS2 cells^43^ (BEI Resources cat# NR-55293) were seeded in 96-well plates at 50 000 cells. The next day, pseudovirus preparations normalized for viral capsid p24 levels (Lenti-X™ GoStix™ Plus) were incubated with dilutions of the indicated antibodies or sera, or media alone for 1 h at 37 °C prior to addition to cells and incubation for 48 h. Cells were then lysed and luciferase activity was assessed using the ONE-Glo™ EX Luciferase Assay System (Promega) according to the manufacturer’s specifications. Detection of relative luciferase units was carried out using a Varioskan Lux plate reader (Thermo Fisher). Percent neutralization was calculated relative to signals obtained in the presence of the virus alone for each experiment.

### Expression and purification of recombinant proteins

Expi293F cells (Thermo Fisher, Cat# A14527) were grown in suspension culture using Expi293 Expression Medium (Thermo Fisher, Cat# A1435102) at 37 °C, 8% CO_2_. Cells were transiently transfected at a density of 3 × 10^6^ cells/mL using linear polyethylenimine (Polysciences Cat# 23966-1). The media was supplemented 24 h after transfection with 2.2 mM valproic acid, and expression was carried out for 3–7 days at 37 °C, 8% CO_2_.

For ectodomain trimer and monomeric human ACE2 (residues 1–615) purification, the supernatant was harvested by centrifugation and filtered through a 0.22-μM filter prior to loading onto a 5 mL HisTrap excel column (Cytiva). The column was washed for 20 CVs with wash buffer (20 mM Tris pH 8.0, 500 mM NaCl), 5 CVs of wash buffer supplemented with 20 mM imidazole, and the protein eluted with elution buffer (20 mM Tris pH 8.0, 500 mM NaCl, 500 mM imidazole). Elution fractions containing the protein were pooled and concentrated (Amicon Ultra 100 kDa cut off for ectodomain, 10 kDa for monomeric ACE2) before gel filtration. Gel filtration was conducted using a Superose 6 10/300 GL column (Cytiva) pre-equilibrated with GF buffer (20 mM Tris pH 8.0, 150 mM NaCl). Peak fractions corresponding to soluble protein were pooled and concentrated to 4.5–5.5 mg/mL (Amicon Ultra 100 kDa cut off for ectodomain, 10 kDa for monomeric ACE2). Protein samples were flash-frozen in liquid nitrogen and stored at −80 °C.

For purification of the RBD and NTD constructs, the supernatant was harvested after 7 days of expression and incubated with 300 µL of Ni-NTA resin (Qiagen) at 4 °C overnight. The resin was washed three times with 5 mL of PBS, then three times with 5 mL of PBS supplemented with 20 mM of imidazole. Proteins were eluted in PBS containing 300 mM of imidazole and then buffer exchanged into PBS and concentrated to 5–10 mg/mL (Amicon Ultra 10 kDa cut off, Millipore Sigma) before flash freezing and storage at −80 °C.

For purification of dimeric human ACE2-FC, the supernatant was harvested after 6 days of expression and flowed through a gravity column containing over 400 µL of Protein A Plus Agarose (Thermo Fisher Cat# 22812) once. The column was washed once with 5 mL of PBS before elution with 0.1 M glycine pH3.5 and immediate neutralization with 1 M Tris pH 8.0. Elutions were pooled and concentrated using an Amicon Ultra 50 kDa cut-off concentrator before gel filtration. Gel filtration was conducted using a Superose 6 10/300 GL column (Cytiva) pre-equilibrated with GF buffer (20 mM Tris pH 8.0, 150 mM NaCl). Peak fractions corresponding to soluble protein were pooled and concentrated to 2–5 mg/mL (Amicon Ultra 50 kDa cut-off). Protein samples were flash-frozen in liquid nitrogen and stored at −80 °C.

### Antibody production

VH-FC ab8, IgG ab1, Fab S309, and Fab S2M11 were produced as previously described^3,13,14^. Plasmids encoding light and heavy chains for Fab 4A8 and Fab 4–8 were synthesized (GeneArt Gene Synthesis, Thermo Fischer Scientific). Heavy chains were designed to incorporate a C terminal 6x histidine tag. Expi293 cells were transfected at a density of 3 × 10^6^ cells/mL using linear polyethylenimine (Polysciences Cat# 23966-1). Twenty-four hours following transfection, media was supplemented with 2.2 mM valproic acid, and expression was carried out for 3–5 days at 37 °C, 8% CO_2_. The supernatant was harvested by centrifugation and filtered through a 0.22 μM filter prior to loading onto a 5 mL HisTrap excel column (Cytiva). The column was washed for 20 CVs with wash buffer (20 mM Tris pH 8.0, 500 mM NaCl), 5 CVs of wash buffer supplemented with 20 mM imidazole. The protein was eluted with elution buffer (20 mM Tris pH 8.0, 500 mM NaCl, 500 mM imidazole). Elution fractions containing the protein were pooled and concentrated (Amicon Ultra 10 kDa cut-off, Millipore Sigma) for gel filtration. Gel filtration was conducted using a Superose 6 10/300 GL column (Cytiva) pre-equilibrated with GF buffer (20 mM Tris pH 8.0, 150 mM NaCl). Peak fractions corresponding to soluble protein were pooled and concentrated to 8–20 mg/mL (Amicon Ultra 10 kDa cut-off, Millipore Sigma). Protein samples were stored at 4 °C until use.

### Electron microscopy sample preparation and data collection

For cryo-EM, S protein samples were prepared at 2.25 mg/mL, with and without the addition of ACE2 or antibody (1:1.25 S protein trimer:ACE2 molar ratio, 1:9 S protein trimer:V_H_ab6 molar ratio, 1:8 S protein trimer:S2M11 fab molar ratio, 1:4 S protein trimer:4A8/4-8 fab molar ratio). Mixtures were incubated on ice for 20 min prior to centrifugation at 14,000 × *g* for 10 min. Vitrified samples of all S protein samples were prepared by first glow discharging Quantifoil R1.2/1.3 Cu mesh 200 holey carbon grids for 30 seconds using a Pelco easiGlow glow discharge unit (Ted Pella) and then applying 1.8 µL of protein suspension to the surface of the grid at a temperature of 10 °C and a humidity level of >98%. Grids were blotted (12 s, blot force −10) and plunge frozen into liquid ethane using a Vitrobot Mark IV (Thermo Fisher Scientific). All cryo-EM samples were imaged using a 300 kV Titan Krios G4 transmission electron microscope (Thermo Fisher Scientific) equipped with a Falcon4 direct electron detector in electron event registration (EER) mode. Movies were collected at ×155,000 magnification (calibrated pixel size of 0.5 Å per physical pixel) over a defocus range of −0.5 µm to −3 μm with a total dose of 40 e^−^/Å^2^ using the EPU automated acquisition software.

### Image processing

The detailed workflow for the data processing is summarized in Supplementary Figs. 1–4, 6–7, 11–14, 17, 23, and 25. In general, all data processing was performed in cryoSPARC v.3.2^44^ unless stated otherwise. Patch mode motion correction (EER upsampling factor 1, EER number of fractions 40), patch mode CTF estimation, reference free particle picking, and particle extraction were carried out on-the-fly in cryoSPARC live. After preprocessing, particles were subjected to 2D classification and/or 3D heterogeneous classification. The final 3D refinement was performed with an estimation of per particle CTF and correction for high-order aberrations.

For the complexes of spike protein ectodomain and human ACE2, focused refinements were performed with a soft mask covering a single RBD and its bound ACE2. For the complexes of spike protein ectodomain and VH ab6, a soft mask covering VH-ab6 and its bound RBD was used in focused refinement. For the complexes of Gamma spike protein ectodomain and fab 4-8/4-A8, another round of 3D refinement with C3 symmetry was carried out, followed by symmetry expansion. The derived particles were then focused-refined with a soft mask covering single NTD and its bound fab.

Global resolution and focused resolution were determined according to the gold-standard FSC^45^.

### Model building and refinement

Initial models either from published coordinates (PDB code 7MJG, 7MJM, 7MJN, 7LXY, 7K43, and 7MJI) or from homology modeling (for 4–8, 4-A8, and V_H_-ab6) were docked into the focused refinement maps or global refinement maps using UCSF Chimera v.1.15^46^. Then, mutation and manual adjustment were performed with COOT v.0.9.3^47^, followed by iterative rounds of refinement in COOT and Phenix v.1.19^48^. Model validation was performed using MolProbity^49^. Figures were prepared using UCSF Chimera, UCSF ChimeraX v.1.1.1^50^, and PyMOL (v.2.2 Schrodinger, LLC).

### Biolayer interferometry (BLI) S protein—ACE2 binding assay

The kinetics of SARS-CoV-2 trimers and human ACE2 binding were analyzed with the biolayer interferometer BLItz (ForteBio, Menlo Park, CA). Protein-A biosensors (ForteBio: 18–5010) were coated with ACE2-mFc (40 µg/mL) for 2 min and incubated in DPBS (pH = 7.4) to establish baselines. Concentrations of 125, 250, 500, and 1000 nM spike trimers were used for the association for 2 min followed by dissociation in DPBS for 5 min. The association (*k*_on_) and dissociation (*k*_off_) rates were derived from the fitting of sensorgrams and used to calculate the binding equilibrium constant (*K*_D_).

### Flow cytometry S protein—ACE2 binding assay

Expi293 cells were transfected with plasmids encoding the various full-length variant S proteins using linear PEI as performed during recombinant spike production. After 3 days of expression, cells were washed with PBS then pelleted and resuspended in incubation buffer (PBS pH 7.4 with 0.02% Tween-20 and 4% BSA) at a concentration of 5 × 10^6^ cells/mL. In all, 100 µl of this mixture was deposited into each well within a 96-well plate followed by a 5 min incubation on ice. Cells were pelleted and resuspended in an incubation buffer containing increasing concentrations of recombinant FC-ACE2 (Sino Biological Cat# 10108-H05H-100) followed by incubation for 20 min on ice. The cells were pelleted and washed with 200 µl of wash buffer (PBS pH 7.4 with 0.02%Tween-20). Next, the cells were incubated in 100 µL of a 1:300 dilution of secondary antibody (Thermo Fisher Cat# A-21235) in an incubation buffer for 10 min. Cells were pelleted and washed twice in 100 µL of wash buffer prior to staining with propidium iodide (Biolegend). Cells were analyzed using a Cytoflex LX at the ubcFLOW cytometry facility. Data were analyzed using FlowJo. The gating strategy employed along with results for un-transfected (negative control) Expi293 cells are available in Supplementary Fig. 10B.

### Enzyme-linked immunosorbent assay (ELISA)

For ELISA, 100 µL of wild-type (D614G), or variant SARS-CoV-2 S proteins, NTDs, and RBDs were coated onto 96-well MaxiSorp™ plates at 2 µg/mL in PBS + 1% casein overnight at 4 °C. All washing steps were performed 3 times with PBS + 0.05% Tween 20 (PBS-T). After washing, wells were incubated with blocking buffer (PBS-T + 1% casein) for 1 h at room temperature. After washing, wells were incubated with dilutions of primary antibodies in PBS-T + 0.5% BSA buffer for 1 h at room temperature. For fab and IgG primary antibodies, wells were incubated with goat anti-human IgG (Jackson ImmunoResearch) at a 1:8000 dilution in PBS-T + 1% casein buffer for 1 h at room temperature. For V_H_ ab6, wells were incubated with mouse anti-FLAG M2 antibody (Sigma Aldrich) at a 1:50 dilution in PBS-T + 1% casein buffer for 1 h at room temperature, washed, and incubated with goat anti-mouse IgG (Thermo Fisher Scientific Cat# 31439) at a 1:5000 dilution in PBS-T + 1% casein buffer for 1 h at room temperature. For ACE2 competition experiments, serial dilutions of V_H_ ab6 were incubated in wells in PBS-T + 1% casein buffer for 1 h at room temperature and washed before incubation with either 0.2 or 1 ng/mL of human ACE2-FC in PBS-T + 0.5% casein for 1 h at room temperature. After washing, wells were incubated with goat anti-human IgG (Jackson ImmunoResearch) at a 1:8000 dilution in PBS-T + 1% casein buffer for 1 h at room temperature. After washing, the substrate solution (Pierce™ 1-Step™) was used for color development according to the manufacturer’s specifications. Optical density at 450 nm was read on a Varioskan Lux plate reader (Thermo Fisher Scientific).

### Surface plasmon resonance (SPR)

SPR experiments were performed on a Biacore T200 instrument. CM5 chips were functionalized with monoclonal anti-Strep-Tag antibody (BIO-RAD Cat# MCA2489) at a concentration of 50 µg/mL for the capture of spike protein ectodomains. After the capture of D614G hexapro ectodomain, either buffer, 175 nM, or 440 nM of V_H_ ab6 was injected onto the chip, followed by 500 nM human ACE2-FC. The surface was regenerated in 10 mM glycine, pH 1 between experiments. The experiments were performed at 25 degrees Celsius, using 10 mM HEPES, 150 mM NaCl, 3 mM EDTA, and 0.05% v/v Surfactant P20 as running buffer. Reference-subtracted curves were utilized for the qualitative assessment of ACE2 competition. Relative response units (RUs) were normalized to a value of 0 immediately before ACE2-FC injection for ease of ACE2-FC binding assessment.

### Authentic SARS-CoV-2 plaque reduction neutralization assay

Neutralization assays were performed using Vero E6 cells (ATCC CRL-1586) that were seeded 24 h prior to the assay in 24-well tissue culture plates at a density of 3 × 10^5^ cells per well. Antibodies were serially diluted twofold (starting concentration of 4, 10, or 40 µg/mL, depending on the antibody being tested) and mixed with an equal volume of 30–50 plaque-forming units of SARS-CoV-2. This results in a final antibody concentration of 2, 5, or 20 µg/mL in the antibody–virus mixture. The following SARS-CoV-2 variants were used: isolate USA-WA1/2020 (NR-52281, BEI Resources); isolate hCoV-19/South Africa/KRISP-EC-K005321/2020 (NR-54008, BEI Resources); isolate hCoV-19/USA/CA_UCSD_5574/2020 (NR-54008, BEI Resources); and isolate hCoV-19/USA/PHC658/2021 (NR-55611, BEI Resources). The antibody-virus mixture was then incubated at 37 °C in a 5% CO_2_ incubator for 1 h and added to the Vero E6 cell seeded monolayers, in duplicate. Plates were then incubated for 1 h at 37 °C in a 5% CO_2_ incubator. Following incubation, an overlay media with 1% agarose-containing media (2× Minimal Essential Medium, 7.5% bovine albumin serum, 10 mM HEPES, 100 µg/mL penicillin G, and 100 U/mL streptomycin) was added to the monolayers. The plates were incubated for 48–72 h (depending on the SARS-CoV2 variant) and then cells were fixed with formaldehyde for 2 h. Following fixation, agar plugs were removed, and cells were stained with crystal violet. In order to assess the input virus, a viral back-titration was performed using a culture medium as a replacement for the antibodies. To estimate the neutralizing capability of each antibody, IC50s were calculated by non-linear regression using the sigmoidal dose–response equation in GraphPad Prism 9. All assays were performed in the University of Pittsburgh Regional Biocontainment Laboratory BSL-3 facility.

### Reporting summary

Further information on research design is available in the Nature Research Reporting Summary linked to this article.

## Supplementary information

Supplementary Information

Reporting Summary

## Data Availability

The data that support this study are available from the corresponding author upon request. The atomic models and cryo-EM density maps have been deposited into the Protein Data Bank (PDB) and Electron Microscopy Data Bank (EMDB) as follows: Alpha (B.1.1.7) apo spike protein: PDB 8DLI and EMD-27502, Alpha (B.1.1.7) spike protein-ACE2 complex (global): PDB 8DLJ and EMD-27503, Alpha (B.1.1.7) spike protein-ACE2 complex (focused): PDB 8DLK and EMD-27504, Beta (B.1.351) apo spike protein: PDB 8DLL and EMD-27505, Beta (B.1.351) spike protein-ACE2 complex (global): PDB 8DLM and EMD-27506, Beta (B.1.351) spike protein-ACE2 complex (focused): PDB 8DLN and EMD-27507, Gamma (P.1) apo spike protein: PDB 8DLO and EMD-27508, Gamma (P.1) spike protein-ACE2 complex (global): PDB 8DLP and EMD-27509, Gamma (P.1) spike protein-ACE2 complex (local): PDB 8DLQ and EMD-27510, Gamma (P.1) spike protein-4-8 complex (global): EMD-27511, Gamma (P.1) spike protein-4-8 complex (focused): PDB 8DLR and EMD-27512, Gamma (P.1) spike protein-4A8 complex (global): EMD-27513, Gamma (P.1) spike protein-4A8 complex (focused): PDB 8DLS and EMD-27514,Epsilon (B.1.429) apo spike protein: PDB 8DLT and EMD-27515, Epsilon (B.1.429) spike protein-ACE2 complex (global): PDB 8DLU and EMD-27516, Epsilon (B.1.429) spike protein-ACE2 complex (focused): PDB 8DLV and EMD-27517, Epsilon (B.1.429) spike protein-S2M11 complex: PDB 8DLW and EMD-27518, Epsilon (B.1.429) spike protein-V_H_ ab6 complex (global): PDB 8DLX and EMD-27519, Epsilon (B.1.429) spike protein-V_H_ ab6 complex (focused): PDB 8DLY and EMD-27520, D614G spike protein-VH ab6 complex (global): PDB 8DLZ and EMD-27521, D614G spike protein-VH ab6 complex (focused): PDB 8DM0 and EMD-27522. Source data are provided with this paper.

## Source data

Source Data
