# Supplementary material for: SARS-CoV-2 variants of concern: spike protein mutational analysis and epitope for broad neutralization
Source: Nat Commun. 2022 Aug 18;13:4696. doi: 10.1038/s41467-022-32262-8 (PMC9388680; doi:10.1038/s41467-022-32262-8)
Supplement: Supplementary file 1 — Supplementary Information [file 41467_2022_32262_MOESM1_ESM.pdf]

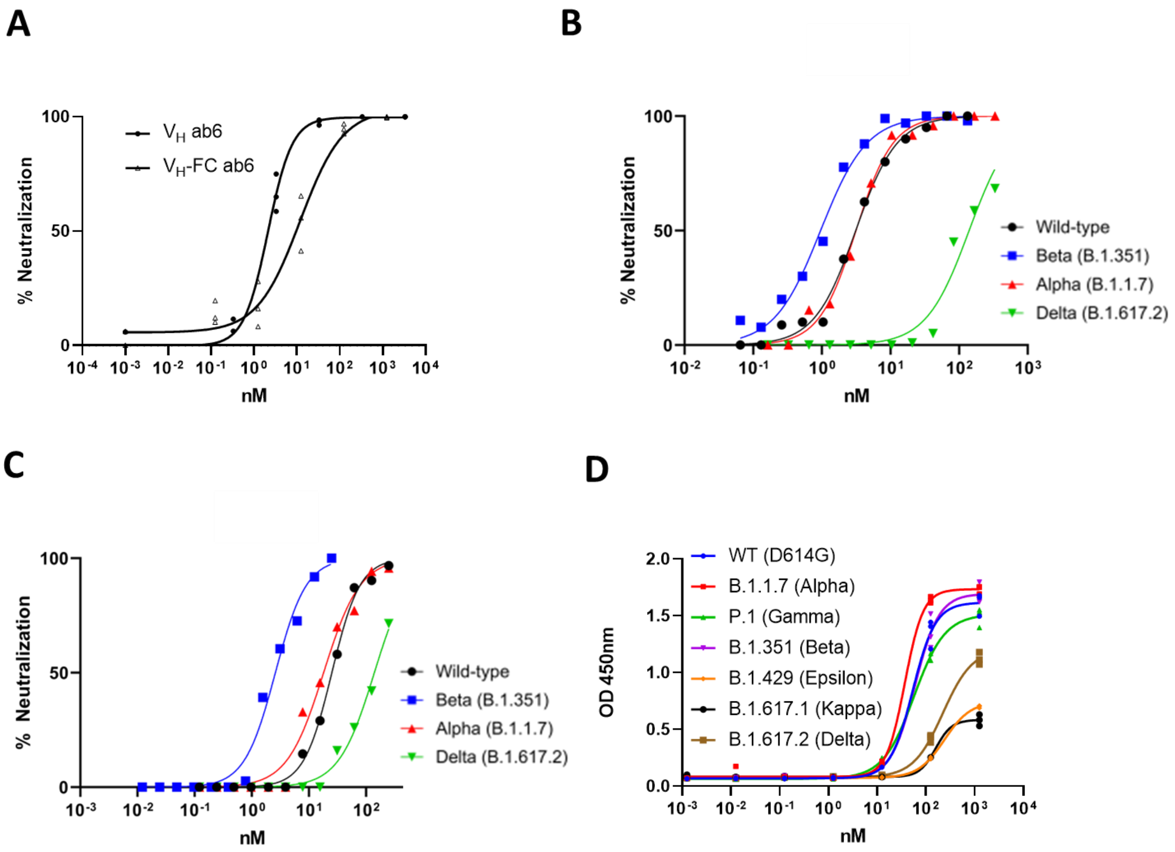

Supplemental Fig. 1. Neutralization and Spike protein binding of SARS-CoV-2 Variants. (A) Pseudovirus neutralization of wild-type spike pseudotyped particles by V_H ab6 and V_H-Fc ab6. **(B-C)** Live virus neutralization assays using ab6**.** Either V_H ab6 (B) or V_H-FC ab6 (C) was used in the plaque reduction neutralization test - PRNT. **(D)** ELISA S protein binding of SARS-CoV-2 variants by VH ab6. ELISAs and pseudoviral neutralization assays were performed in technical triplicate (n=3) and are shown as individual points. PRNT experiments were performed in duplicate, and the mean is plotted. The pseudovirus neutralization utilized the D614G wild-type, whereas the PRNT utilized the USA-WA1/2020 isolate as wild-type. Source data are provided as a Source Data file.

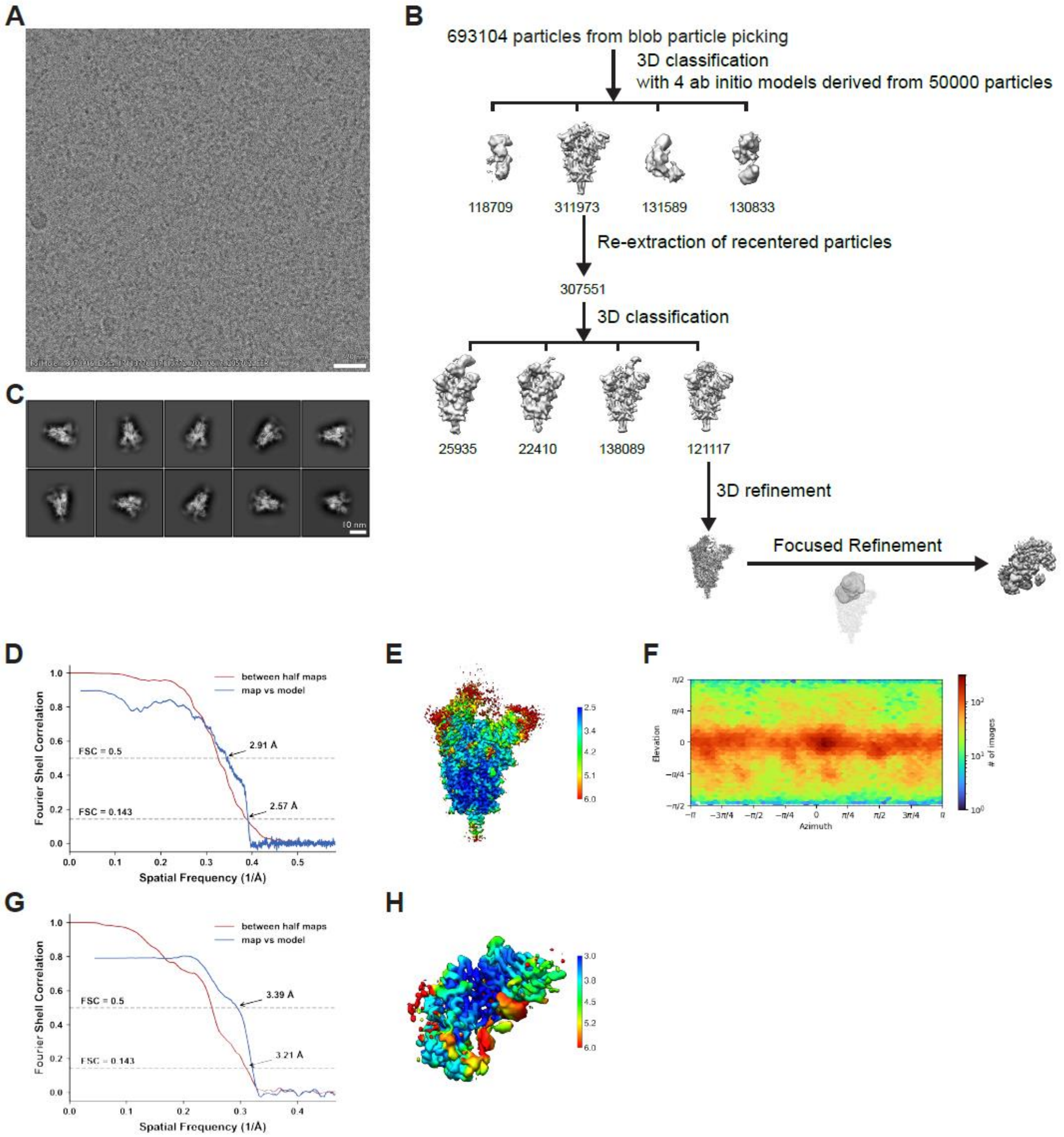

Supplemental Fig. 2. Cryo-EM data processing and validation for complex of D614G spike protein ectodomain and V_H-ab6. (A) Representative cryo-EM micrograph. **(B)** Workflow of cryo-EM image processing. **(C)** Representative 2D classes. **(D-F)** FSC curves (D), local resolution (E) and viewing direction distribution plot (F) of global refinement. **(G-H)** FSC curves (G) and local resolution (H) of focused refinement.

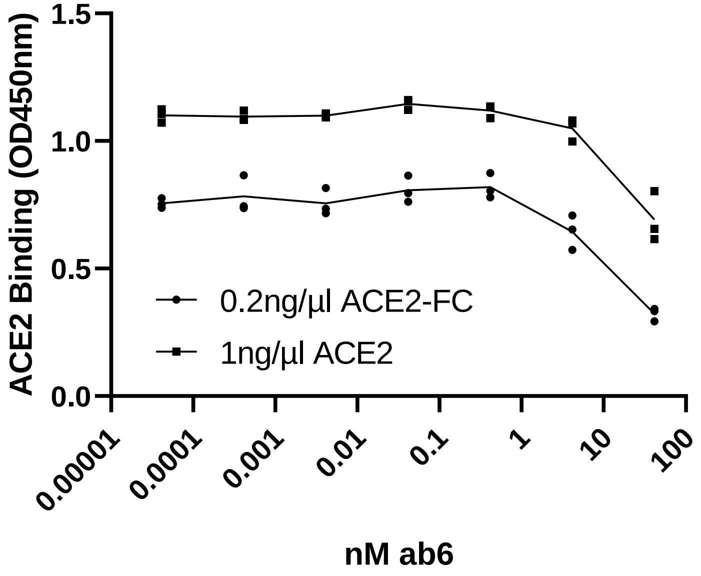

Supplemental Fig. 3. ELISA based ACE2 competition assay. The ability of V_H ab6 to compete with the indicated concentrations of ACE2-FC was assessed via competition ELISA experiments. Experiments were performed in 3 technical replicates (n=3) which are shown as points. Source data are provided as a Source Data file.

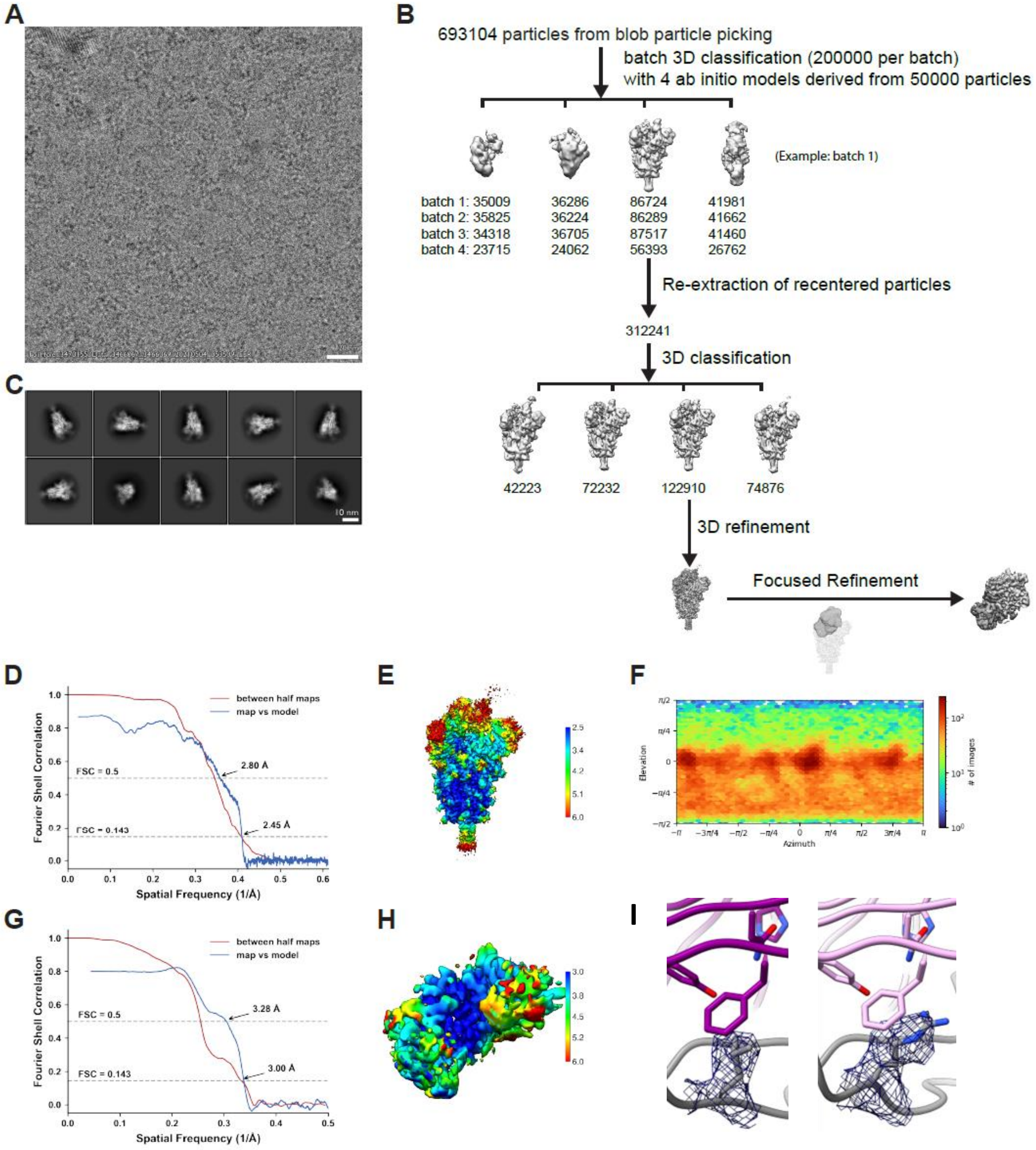

Supplemental Fig. 4. Cryo-EM data processing, validation for complex of Epsilon spike protein ectodomain and V_H-ab6, and visualization of position 452 within WT and Epsilon spike protein ectodomains when bound by V_H-ab6 . (A) Representative cryo-EM micrograph. **(B)** Workflow of cryo-EM image processing. **(C)** Representative 2D classes. **(D-F)** FSC curves (D), local resolution (E) and viewing direction distribution plot (F) of global refinement. **(G-H)** FSC curves (G) and local resolution (H) of focused refinement. **(I)** Visualization of CryoEM map density at position 452 within the WT-V_H-ab6 (Left) and Epsilon-V_H-ab6 complexes (Right), related to Figure 1i.

A

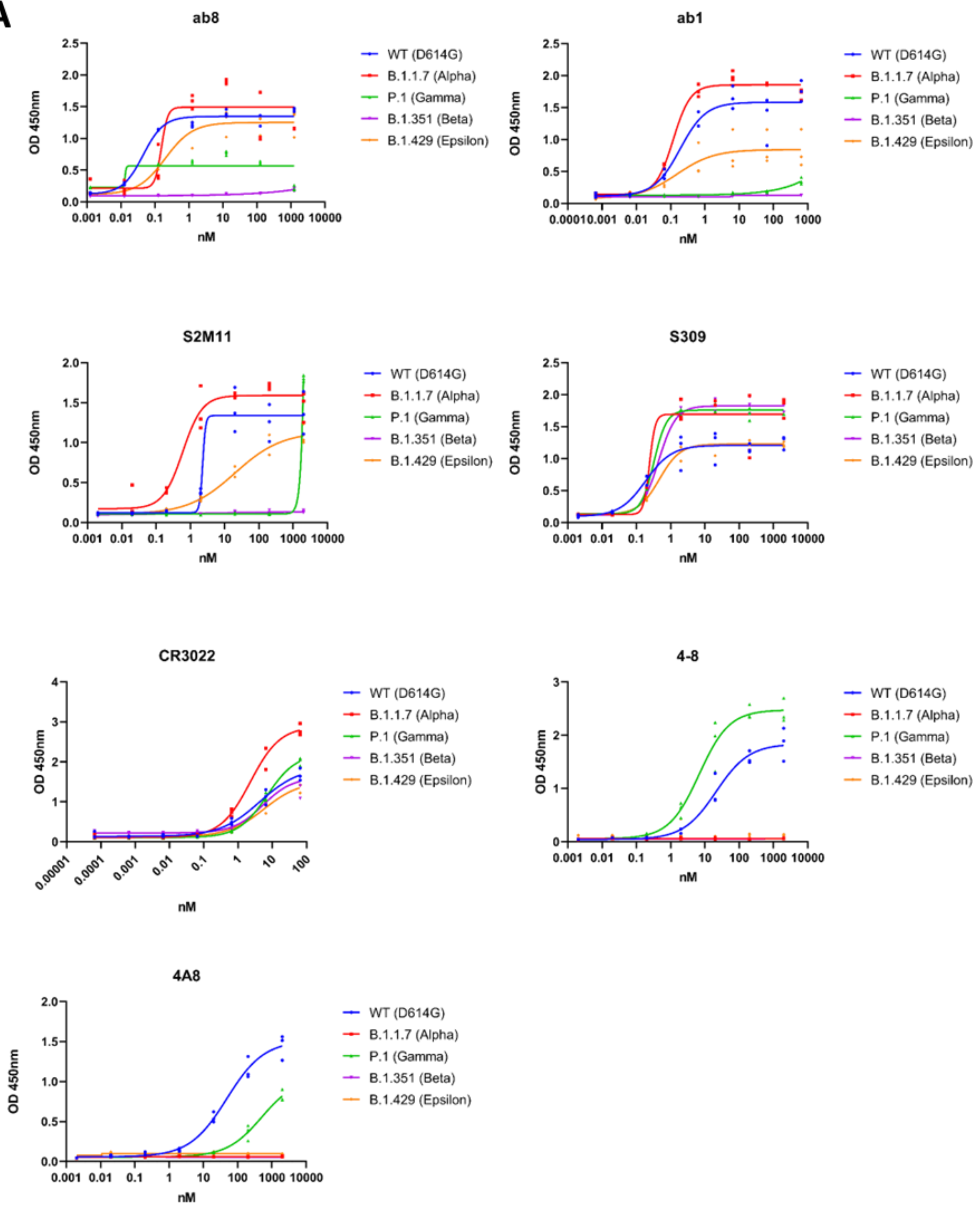

ab8
ab1
S2M11
S309
CR3022
4-8
4A8
OD 450nm
nM
WT (D614G)
B.1.1.7 (Alpha)
P.1 (Gamma)
B.1.351 (Beta)
B.1.429 (Epsilon)

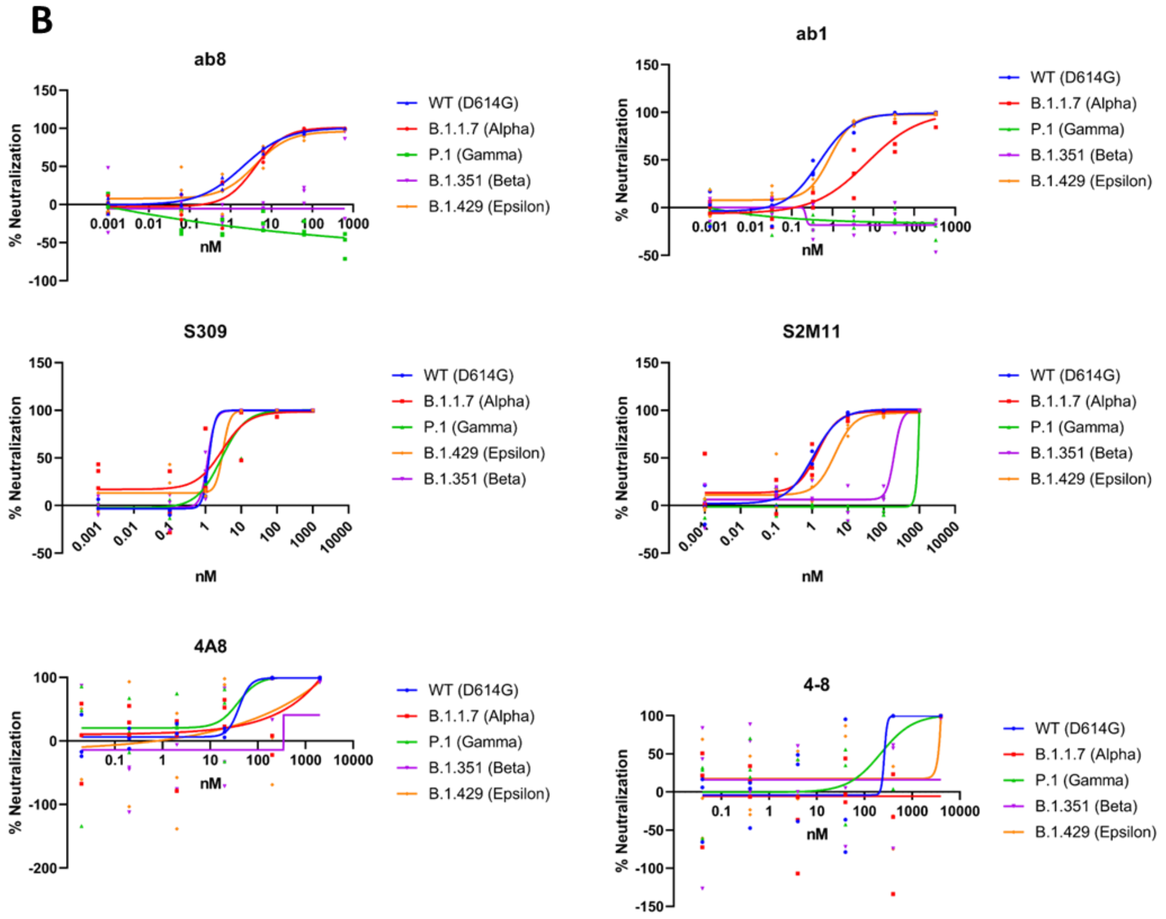

Supplemental Fig. 5. Antibody binding and neutralization curves. Related to Figure 2B. **(A)** Antibody binding curves as determined via ELISA. Experiments were performed in technical triplicate (n=3) and are shown as points. **(B)** Antibody neutralization of variant pseudoviruses, with comparisons to previously published WT (D614G) neutralization data[1]. Experiments were performed in at least technical duplicate (n=2) and are shown as points. Source data are provided as a Source Data file.

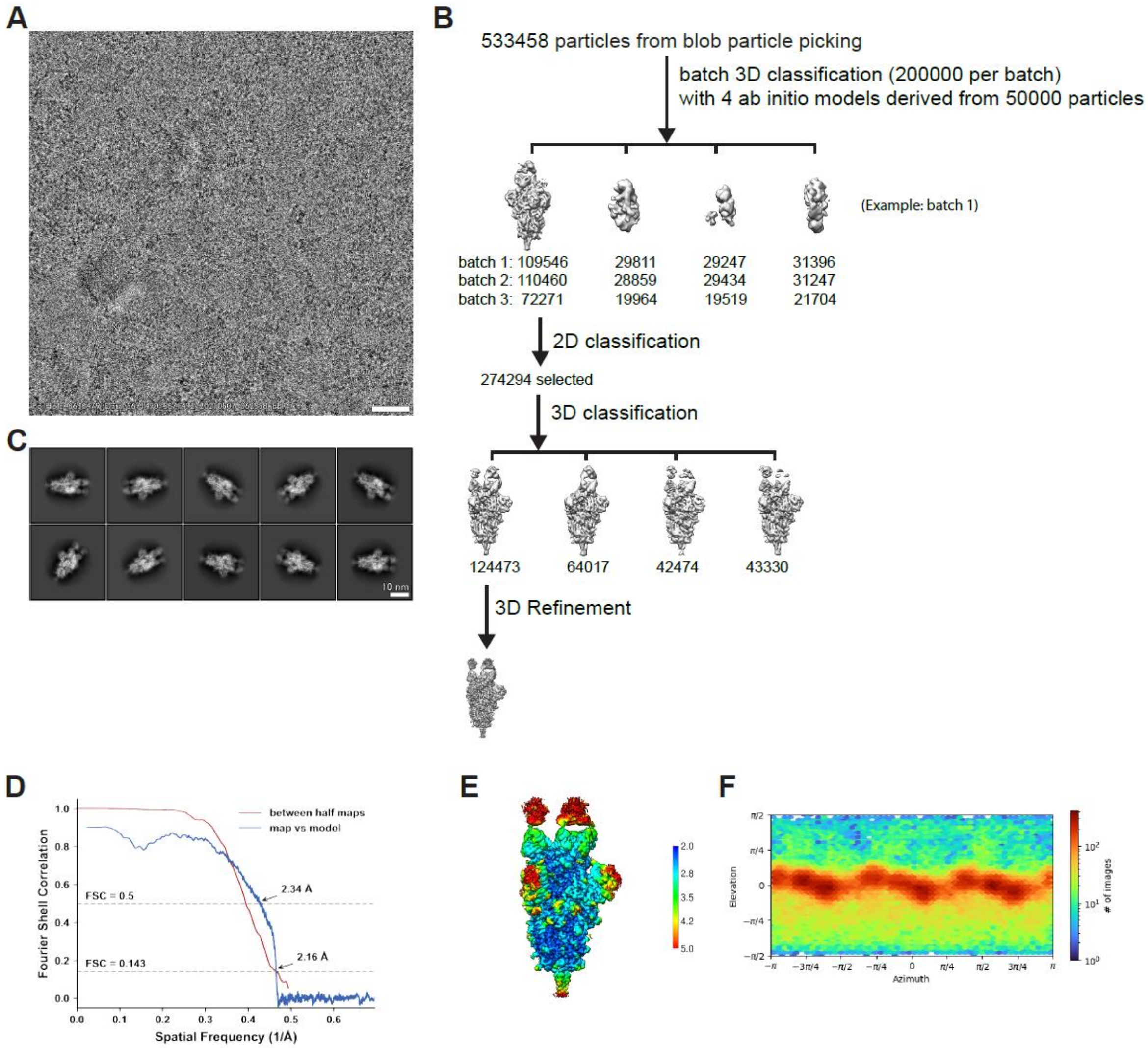

Supplemental Fig. 6. Cryo-EM data processing and validation for complex of Epsilon spike protein ectodomain and S2M11. (A) Representative cryo-EM micrograph. **(B)** Workflow of cryo-EM image processing. **(C)** Representative 2D classes. **(D)** FSC curves. **(E)** Local resolution. **(F)** Viewing direction distribution plot.

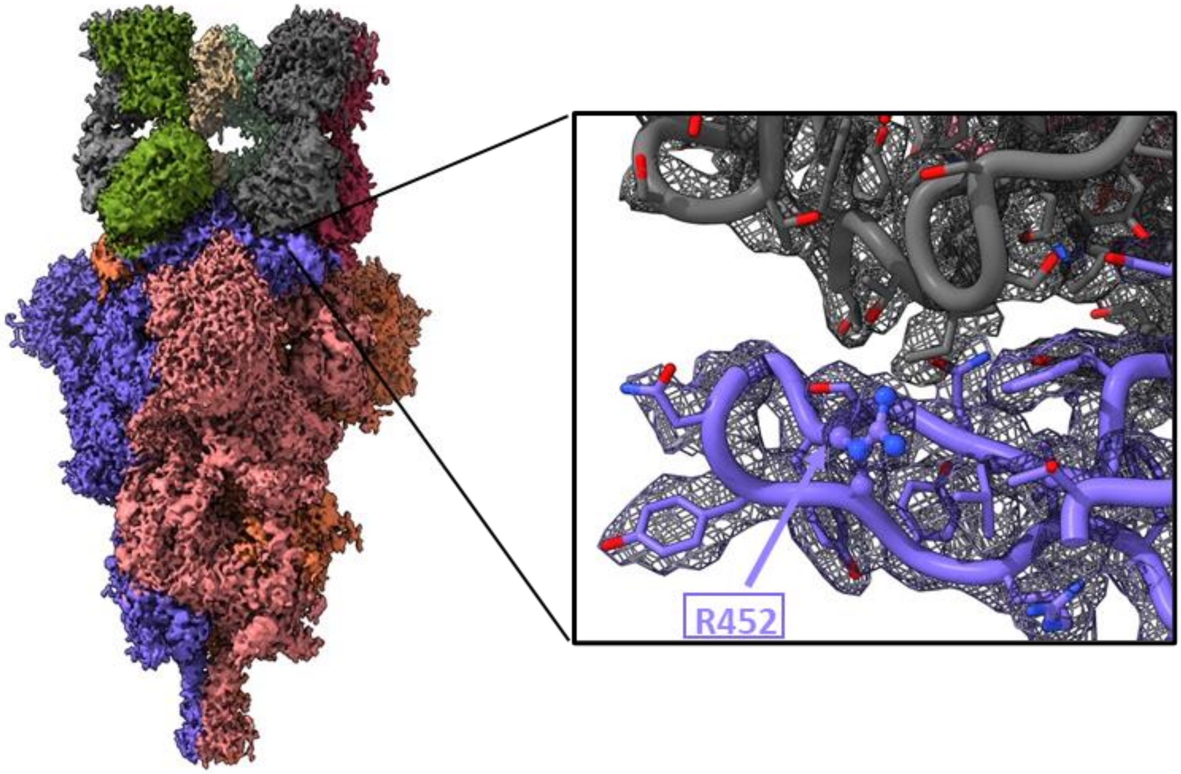

Supplemental Fig. 7. Structure of the Epsilon Variant Spike – S2M11 complex. Global map of the Epsilon Variant Spike – S2M11 complex and zoomed in view of map and model at the S2M11(Grey)-RBD(Purple) interface. R452 is indicated with an arrow and boxed label.

A

Sample ID	Sample Status	Vaccine Dose	Immunization to Serum Draw Time
P0	Vaccine post-COVID19	1st	4 Weeks
P1	Vaccine	1st	3 Weeks
P2	Negative	n/a	n/a
P3	Vaccine	1st	6 Weeks
P4	Negative	n/a	n/a
P5	Vaccine	1st	1 Week
P6	Vaccine	1st	3 Weeks
P7	Negative	n/a	n/a
P8	Vaccine post-COVID19	1st	9 Weeks
P9	Vaccine post-COVID19	1st	7.5 Weeks
P10	Vaccine post-COVID19	1st	6.5 Weeks
P11	Vaccine pre-COVID19	1st	7 Weeks
P12	Vaccine post-COVID19	1st	8.5 Weeks
P13	COVID19	n/a	n/a (2 Weeks post-infection)
14a	Vaccine	2nd	3 Weeks
14b	Vaccine	2nd	4 Weeks
P16	Negative	n/a	n/a
P17	Negative	n/a	n/a
P19	Negative	n/a	n/a

(Rows 14a and 14b bracketed as P14)

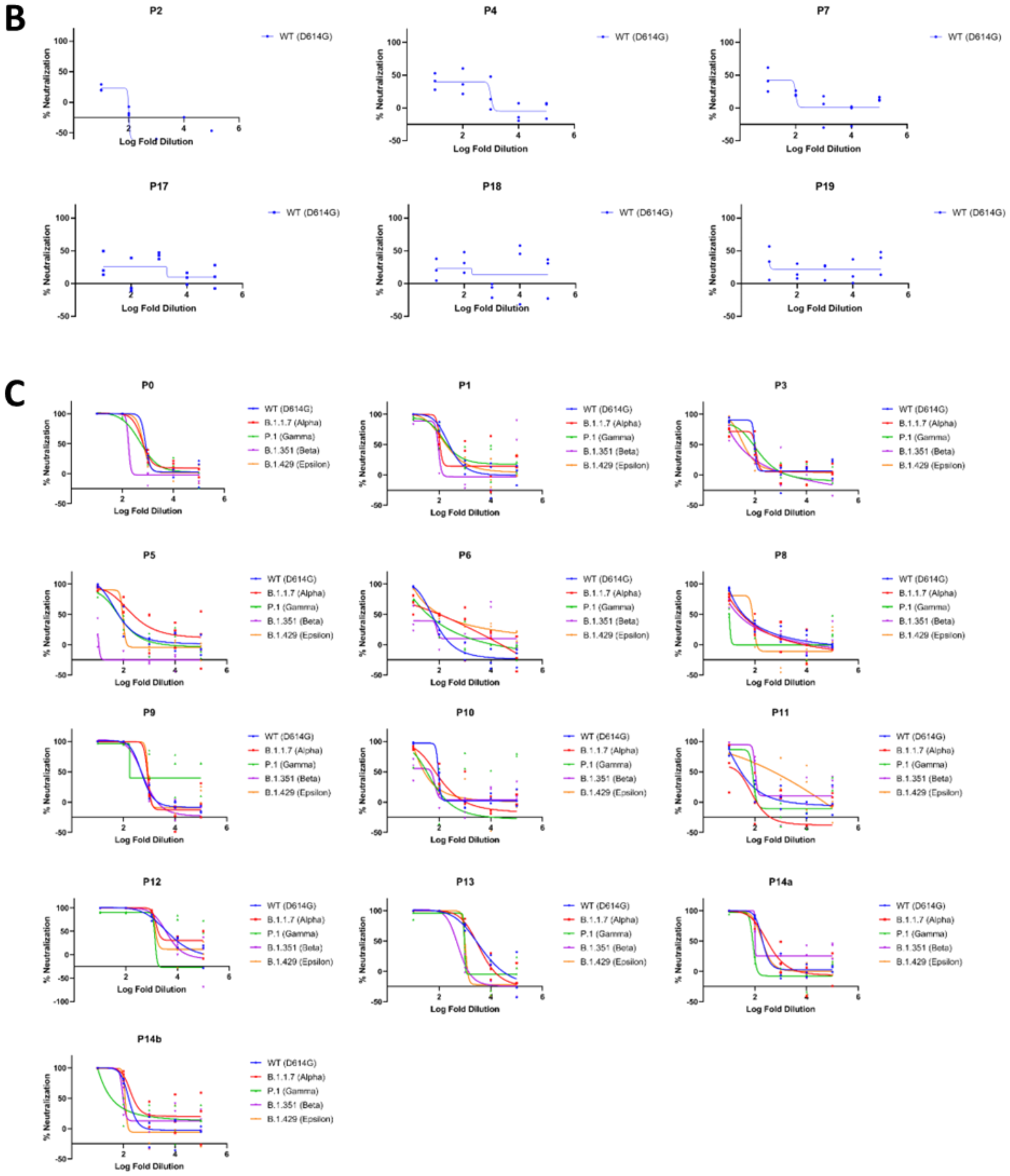

Supplemental Fig. 8. Patient-derived sera sample information and raw pseudovirus neutralization data. Related to figure 2C-D. **(A)** Patient-derived sera sample information including sample number, vaccination status, vaccination dose number and immunization to serum draw time. (n/a: not applicable, Serum for P14 was drawn at 2 time points as indicated by P14a/b). **(B)** Neutralization of wild type spike pseudotyped virus using pre-pandemic sera samples. **(C)** Neutralization of variant pseudoviruses by the indicated patient sera and comparisons to previously determined WT neutralization data[1]. Experiments from the present study were performed in technical triplicate (n=3) and are shown as points. Source data are provided as a Source Data file.

A

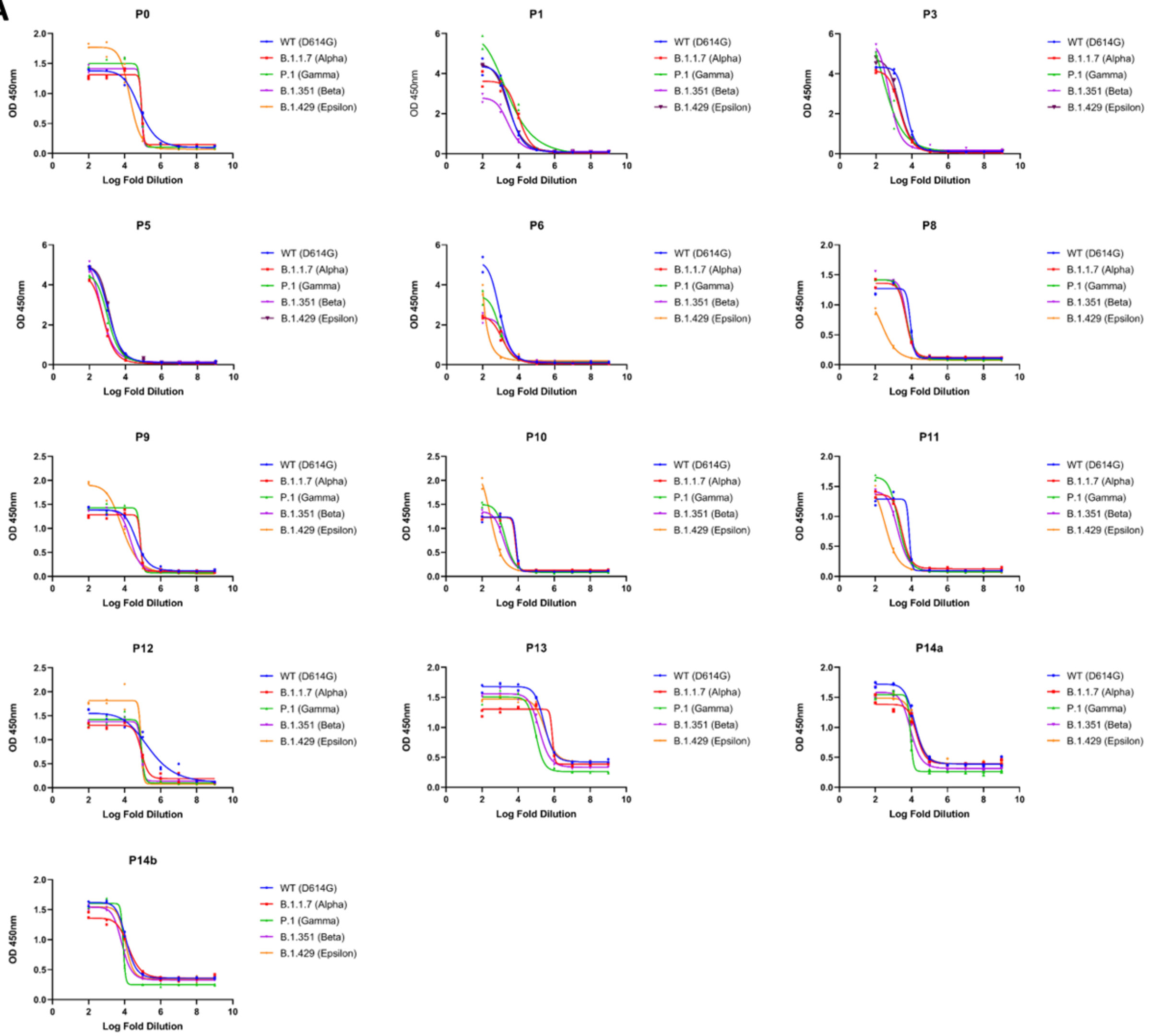
P0
P1
P3
P5
P6
P8
P9
P10
P11
P12
P13
P14a
P14b
OD 450nm
Log Fold Dilution
WT (D614G)
B.1.1.7 (Alpha)
P.1 (Gamma)
B.1.351 (Beta)
B.1.429 (Epsilon)

B

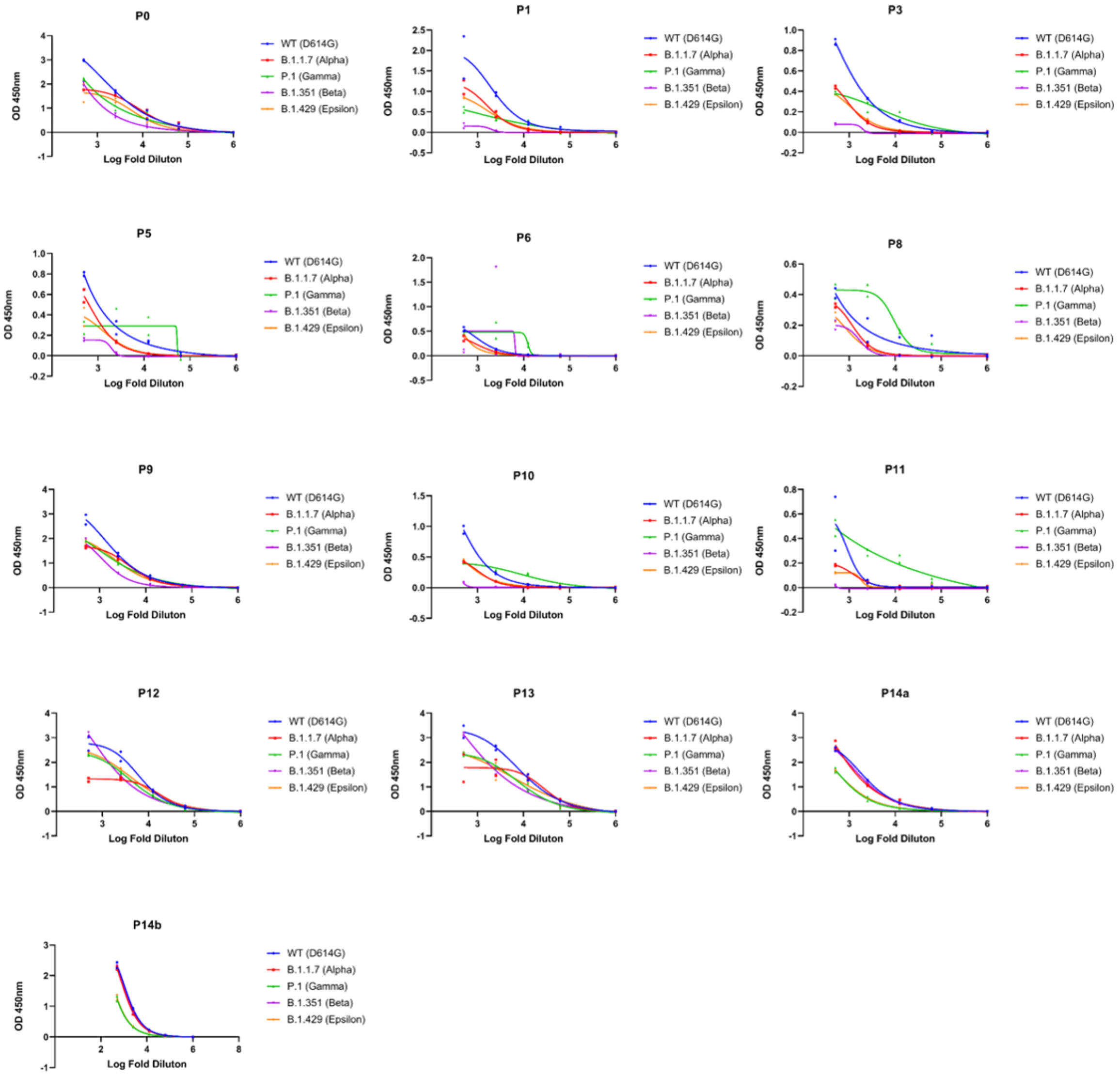
P0
P1
P3
P5
P6
P8
P9
P10
P11
P12
P13
P14a
P14b
WT (D614G)
B.1.1.7 (Alpha)
P.1 (Gamma)
B.1.351 (Beta)
B.1.429 (Epsilon)
OD 450nm
Log Fold Diluton

C

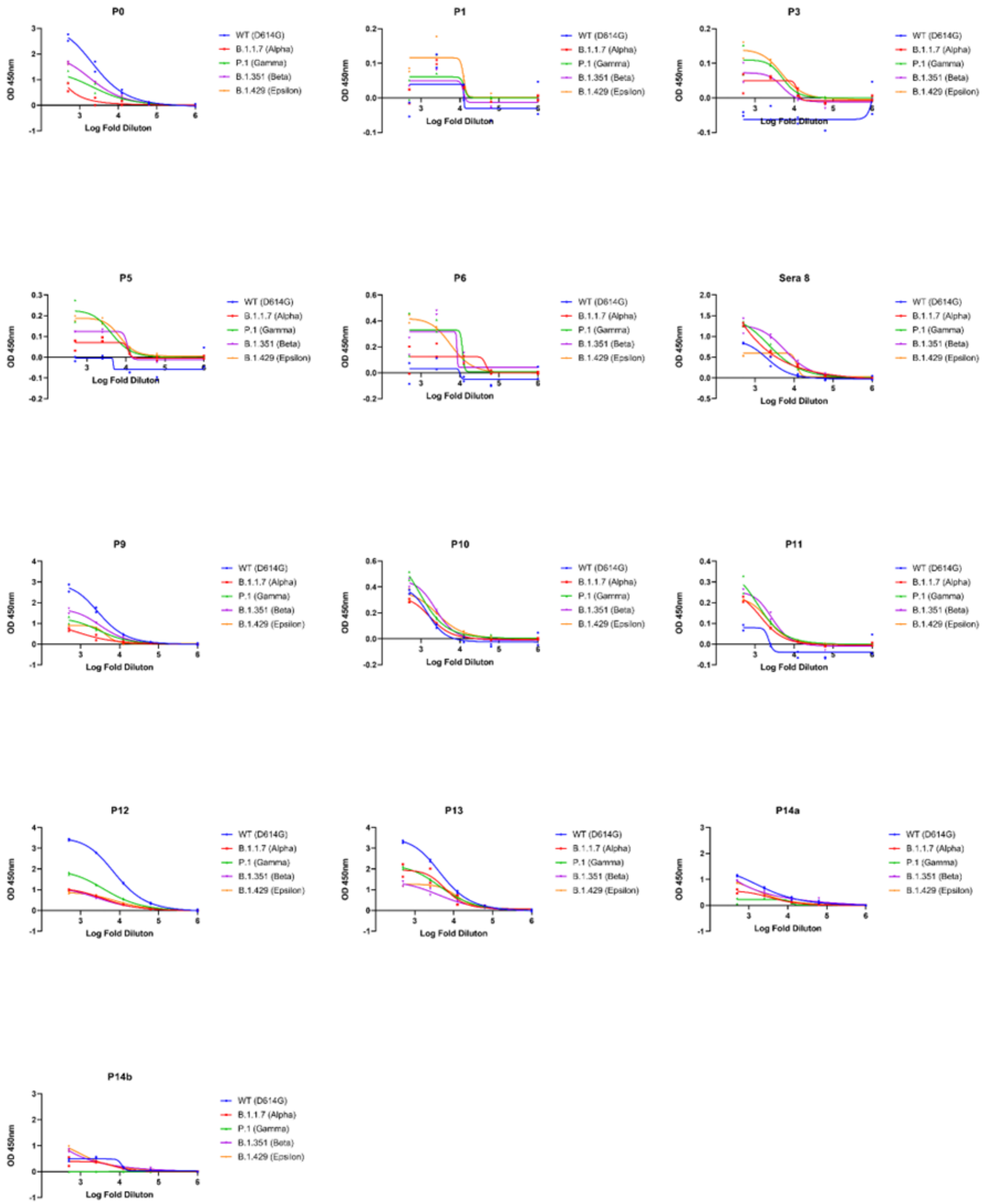
P0
P1
P3
P5
P6
Sera 8
P9
P10
P11
P12
P13
P14a
P14b
OD 450nm
Log Fold Diluton
WT (D614G)
B.1.1.7 (Alpha)
P.1 (Gamma)
B.1.351 (Beta)
B.1.429 (Epsilon)

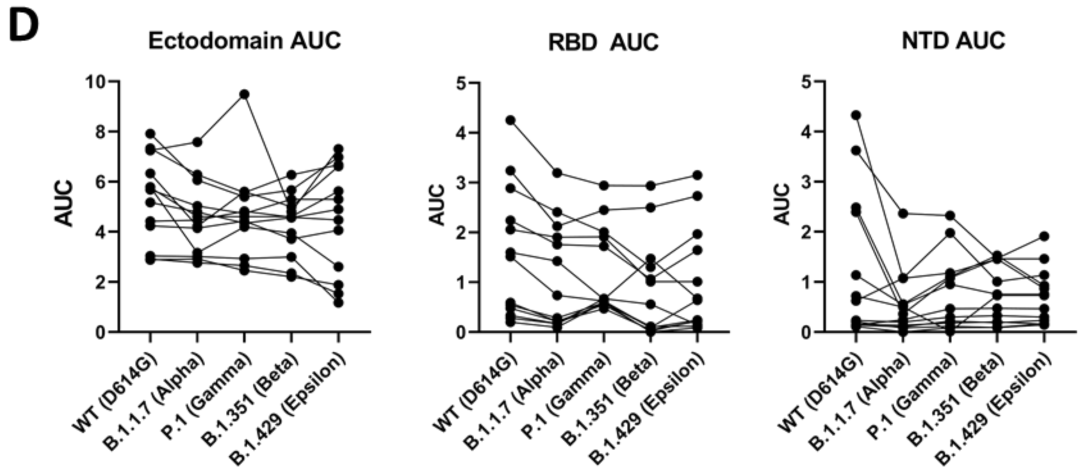

Supplemental Fig. 9. Ectodomain, RBD, and NTD binding by antibodies in patient sera. Related to Figure 2D. Binding of wild type or variant ectodomains **(A)**, RBDs **(B)**, or NTDs **(C)** as assessed by ELISA. Experiments were performed in technical duplicate (n=2), and the results are plotted as points. **(D)** Aggregated area under the curve (AUC) values for each serum sample, protein construct, and spike variant. Source data are provided as a Source Data file.

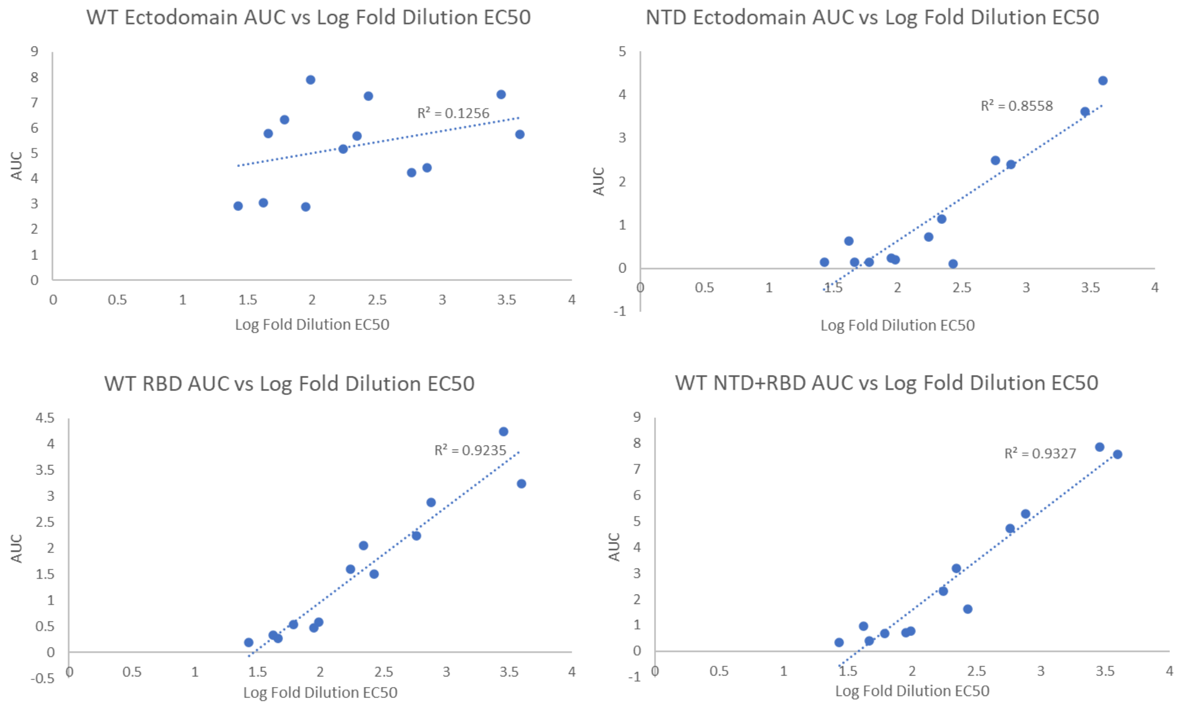

Supplemental Fig. 10. High correlation between NTD and RBD binding antibody levels and pseudoviral neutralization in patient derived sera. Wild type (WT) Ectodomain, NTD, and RBD binding by patient sera was assessed via ELISA and area under the curve (AUC) was calculated from the resulting data. AUC's were correlated with neutralization potencies for wild type spike pseudo typed virus via linear regression. Correlation coefficients are shown for each comparison. Source data are provided as a Source Data file.

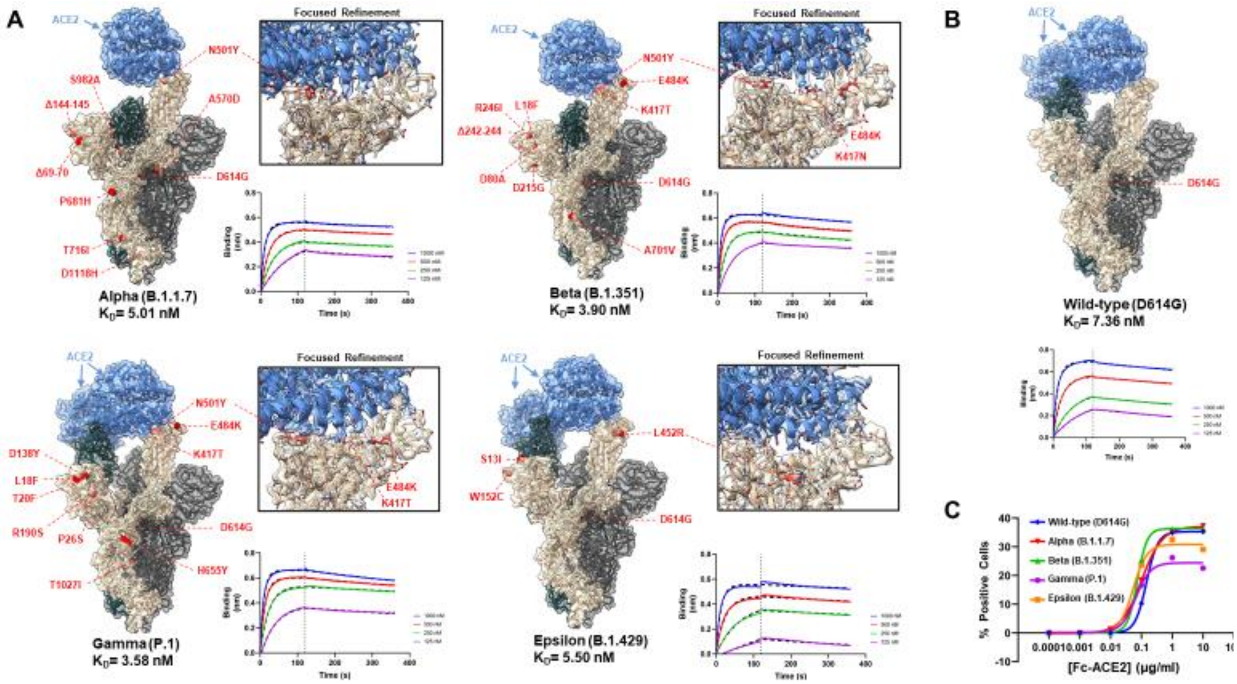

Supplemental Fig. 11. Structural and Functional Analysis of ACE2 Binding by Variant Spikes. (A) CryoEM and biolayer inferometry (BLI) analysis of ACE2 binding by variant spikes. Shown for each variant are global spike-ACE2 complex models with mutational positions highlighted as red spheres (locations of mutations which can not be modelled are approximated as occurring at the nearest modelled residue), maps and models of the ACE2-RBD interface obtained via focused refinement, and BLI sensorgrams for ACE2-spike binding experiments. **(B)** Global spike-ACE2 complex model and BLI sensorgram for the wild-type spike. **(C)** ACE2 binding of cells expressing full-length spikes was measured by Flow-cytometry. Source data are provided as a Source Data file.

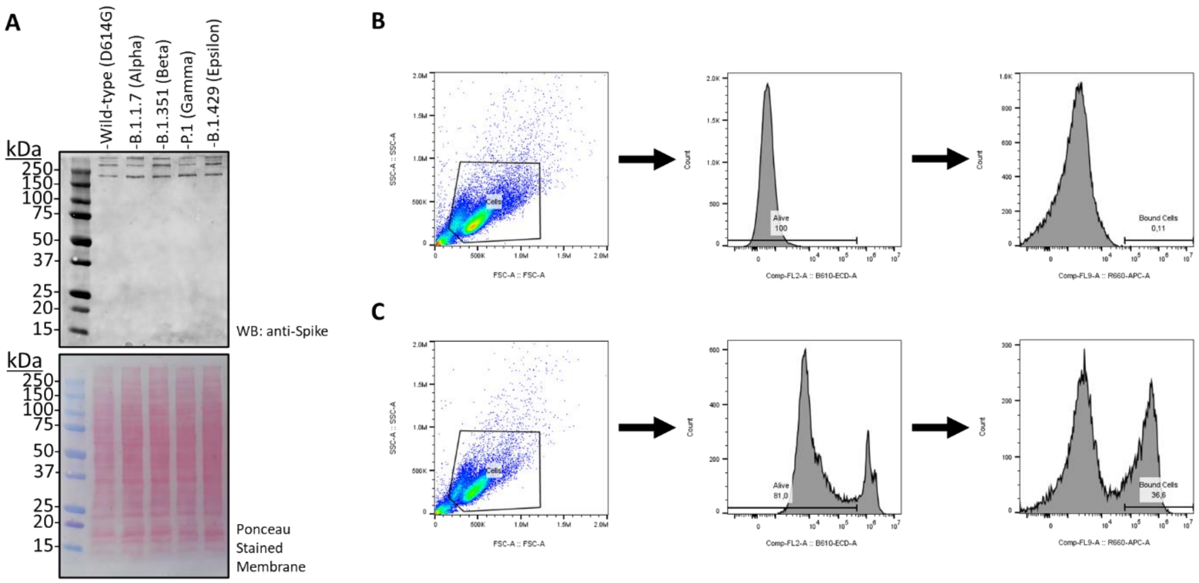

Supplemental Fig. 12. Full length spike protein expression and flow cytometry gating strategy. (A) Western blot of full-length spike proteins expressed in Expi293 cells. The ponceau stained membrane is shown as a loading control. This experiment was performed once. **(B-C)** Gating strategy used for flow cytometry experiments. Panel (B) depicts signals obtained for un-transfected Expi293 cells incubated with the highest concentration of ACE2 utilized (10µg/ml) and panel (C) depicts signals obtained for spike expressing Expi293 cells under the same conditions. Source data are provided as a Source Data file.

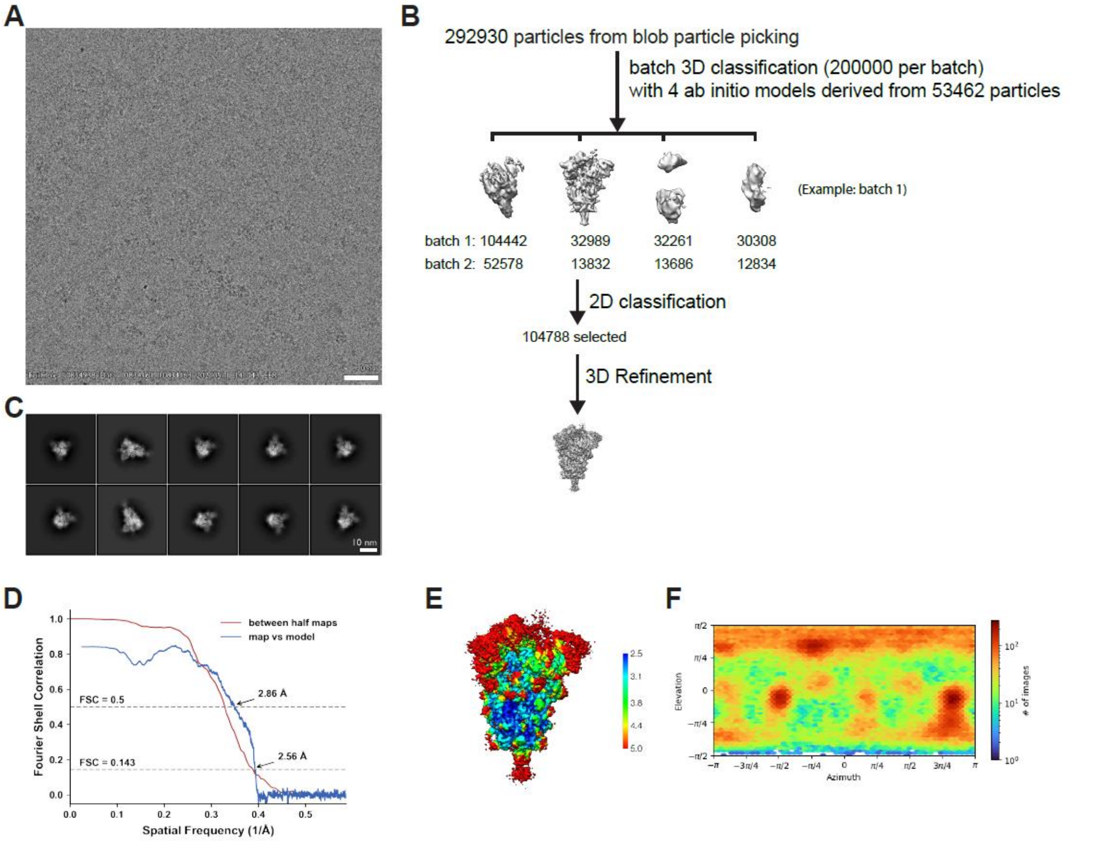

Supplemental Fig. 13. Cryo-EM data processing and validation for the Alpha spike protein ectodomain. **(A)** Representative cryo-EM micrograph. **(B)** Workflow of cryo-EM image processing. **(C)** Representative 2D classes. **(D)** FSC curves. **(E)** Local resolution. **(F)** Viewing direction distribution plot.

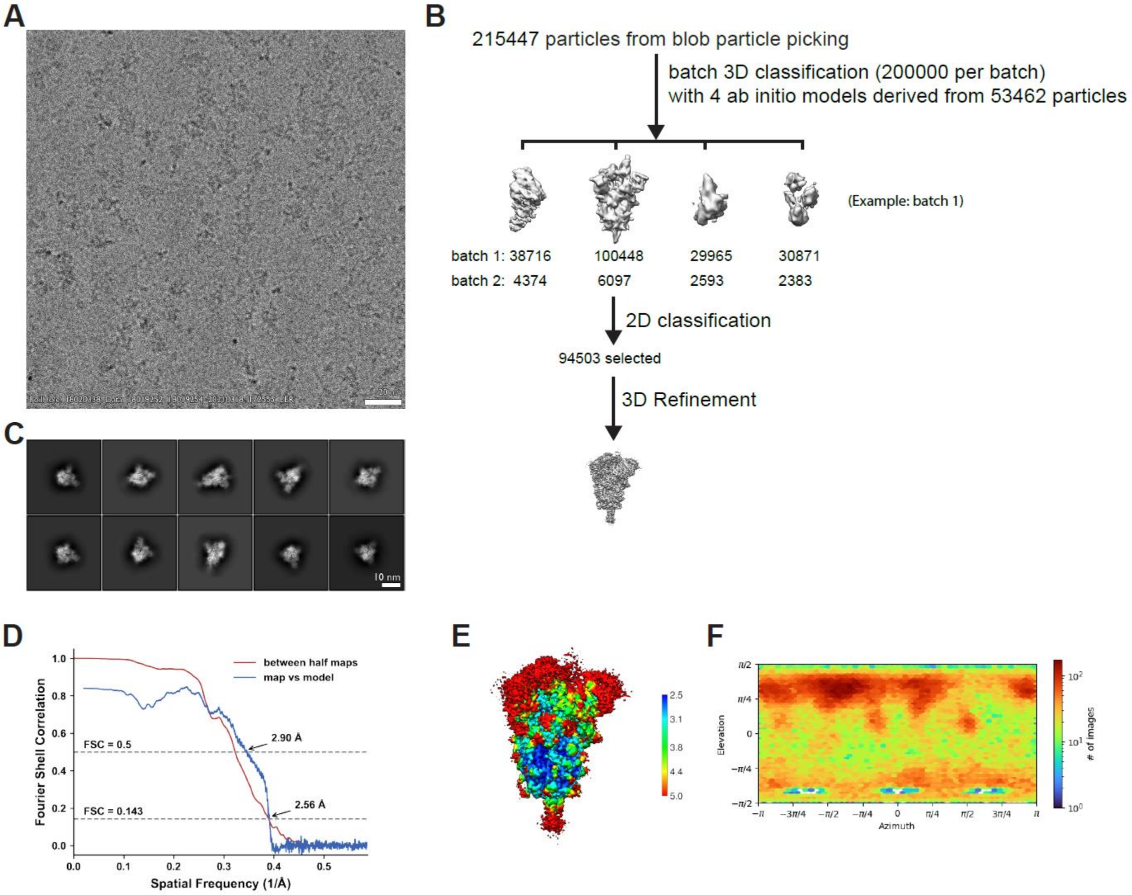

Supplemental Fig. 14. Cryo-EM data processing and validation for the Beta spike protein ectodomain. **(A)** Representative cryo-EM micrograph. **(B)** Workflow of cryo-EM image processing. **(C)** Representative 2D classes. **(D)** FSC curves. **(E)** Local resolution. **(F)** Viewing direction distribution plot.

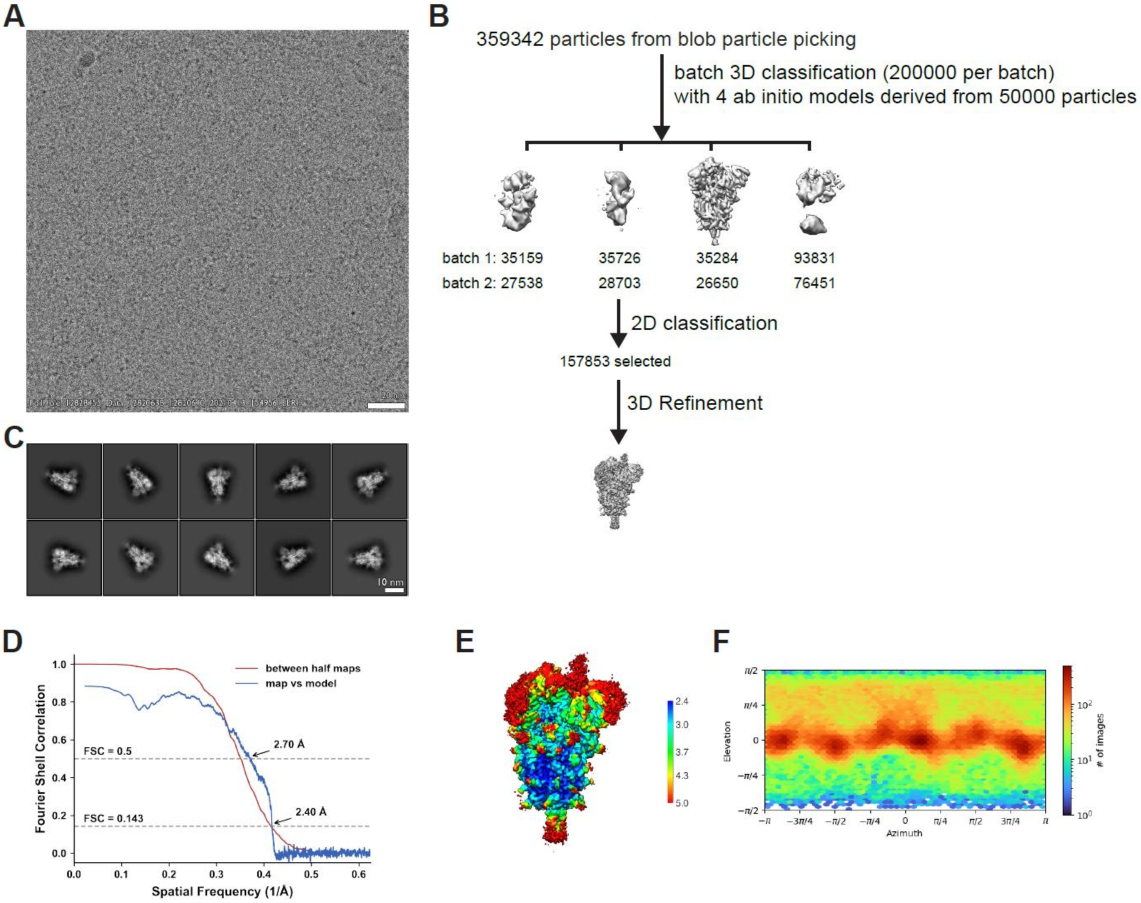

Supplemental Fig. 15. Cryo-EM data processing and validation for the Epsilon spike protein ectodomain. **(A)** Representative cryo-EM micrograph. **(B)** Workflow of cryo-EM image processing. **(C)** Representative 2D classes. **(D)** FSC curves. **(E)** Local resolution. **(F)** Viewing direction distribution plot.

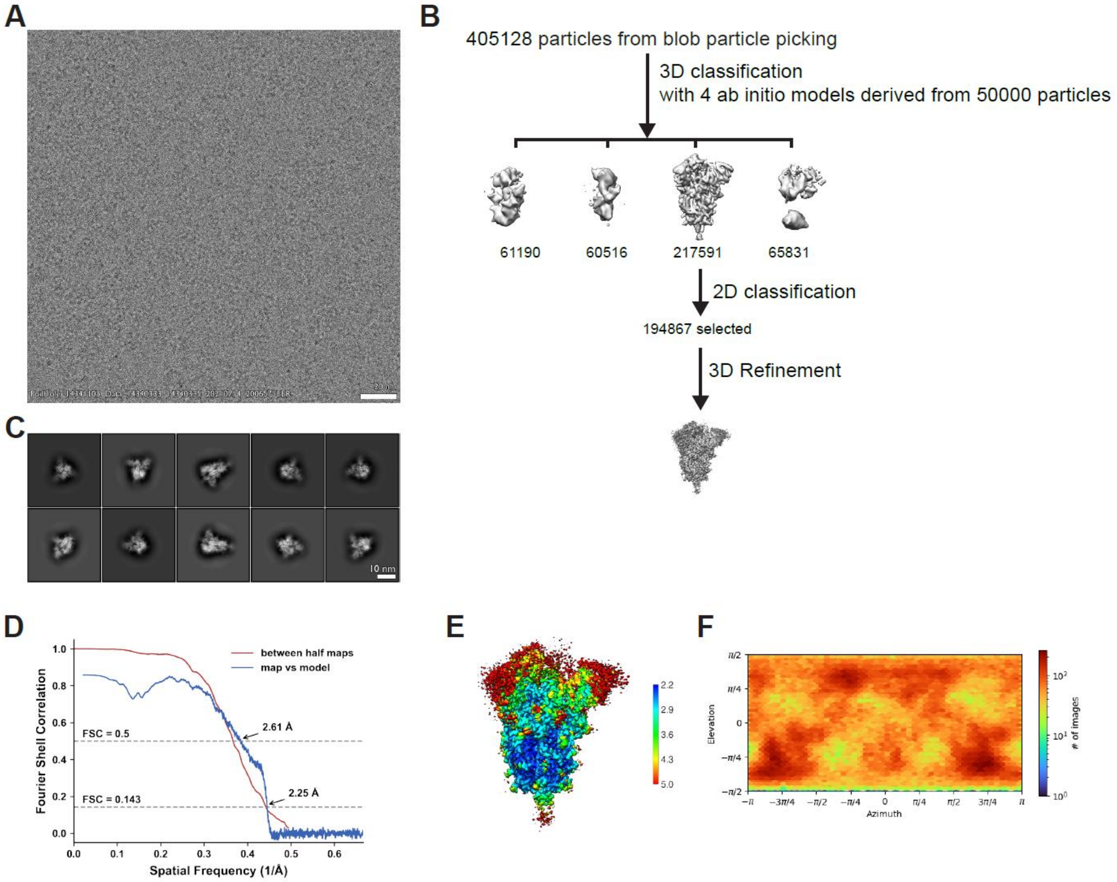

Supplemental Fig. 16. Cryo-EM data processing and validation for the Gamma spike protein ectodomain. **(A)** Representative cryo-EM micrograph. **(B)** Workflow of cryo-EM image processing. **(C)** Representative 2D classes. **(D)** FSC curves. **(E)** Local resolution. **(F)** Viewing direction distribution plot.

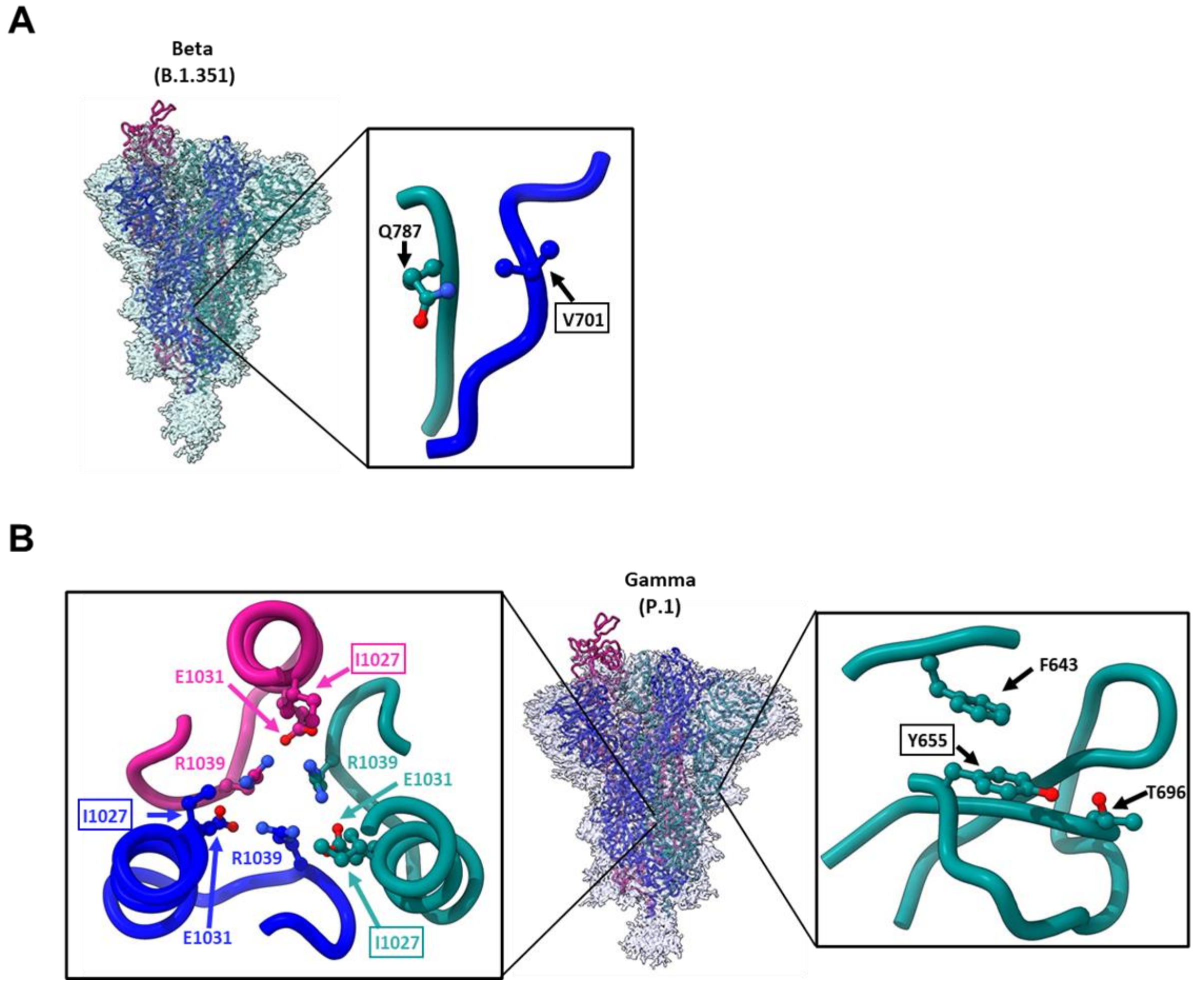

Supplemental Fig. 17. Structural impacts of S2 mutations within the Beta and Gamma S protiens. (A) Global map and model of the Beta variant spike along with a zoomed in view of the A701->V mutation. **(B)** Global map and model of the Gamma variant spike along with zoomed in views of the T1027->I and H655->Y mutations. Mutated residues are indicated as boxed labels.

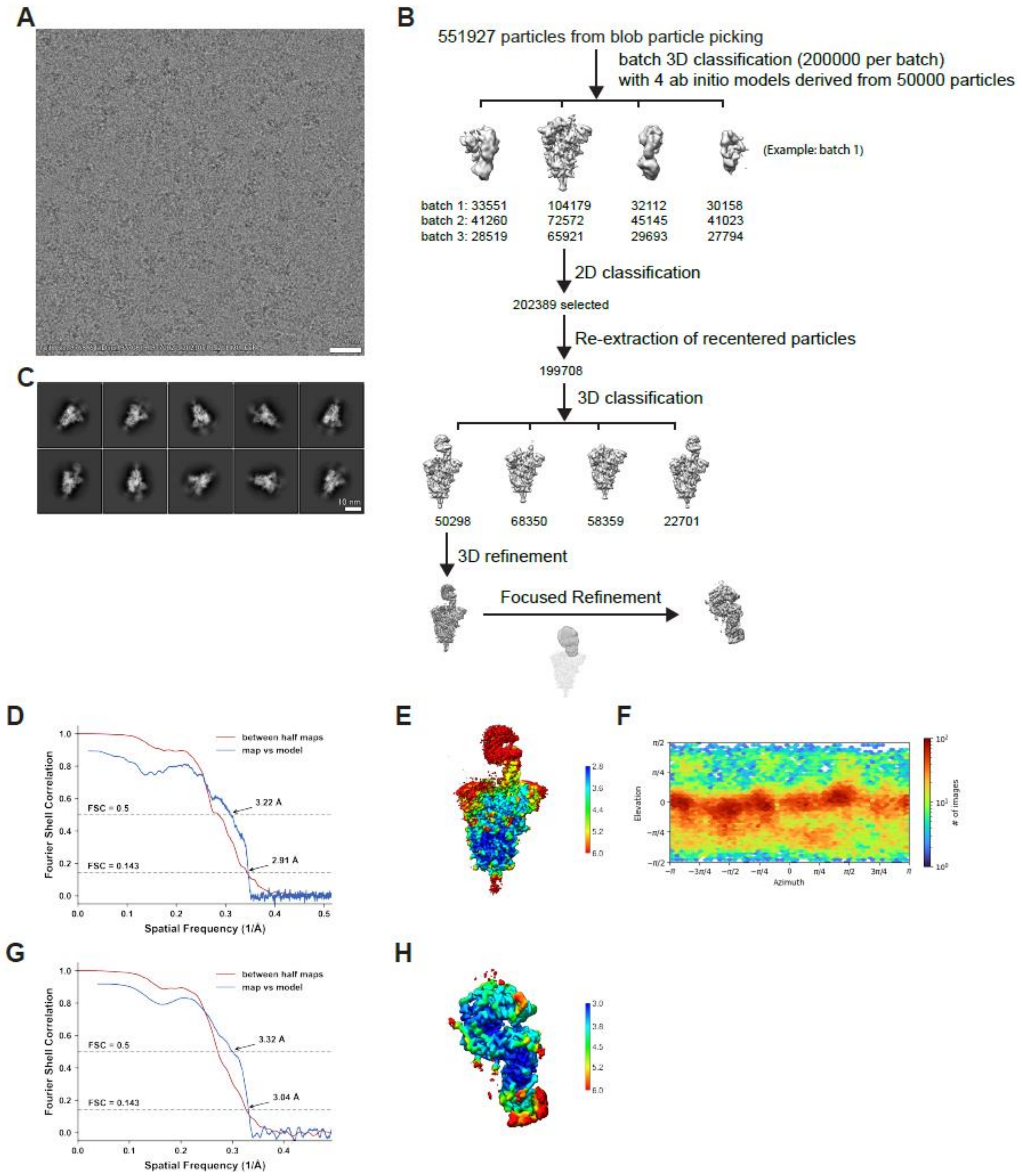

Supplemental Fig. 18. Cryo-EM data processing and validation for complex of Alpha spike protein ectodomain and human ACE2. (A) Representative cryo-EM micrograph. **(B)** Workflow of cryo-EM image processing. **(C)** Representative 2D classes. **(D-F)** FSC curves (D), local resolution (E) and viewing direction distribution plot (F) of global refinement. **(G-H)** FSC curves (G) and local resolution (H) of focused refinement.

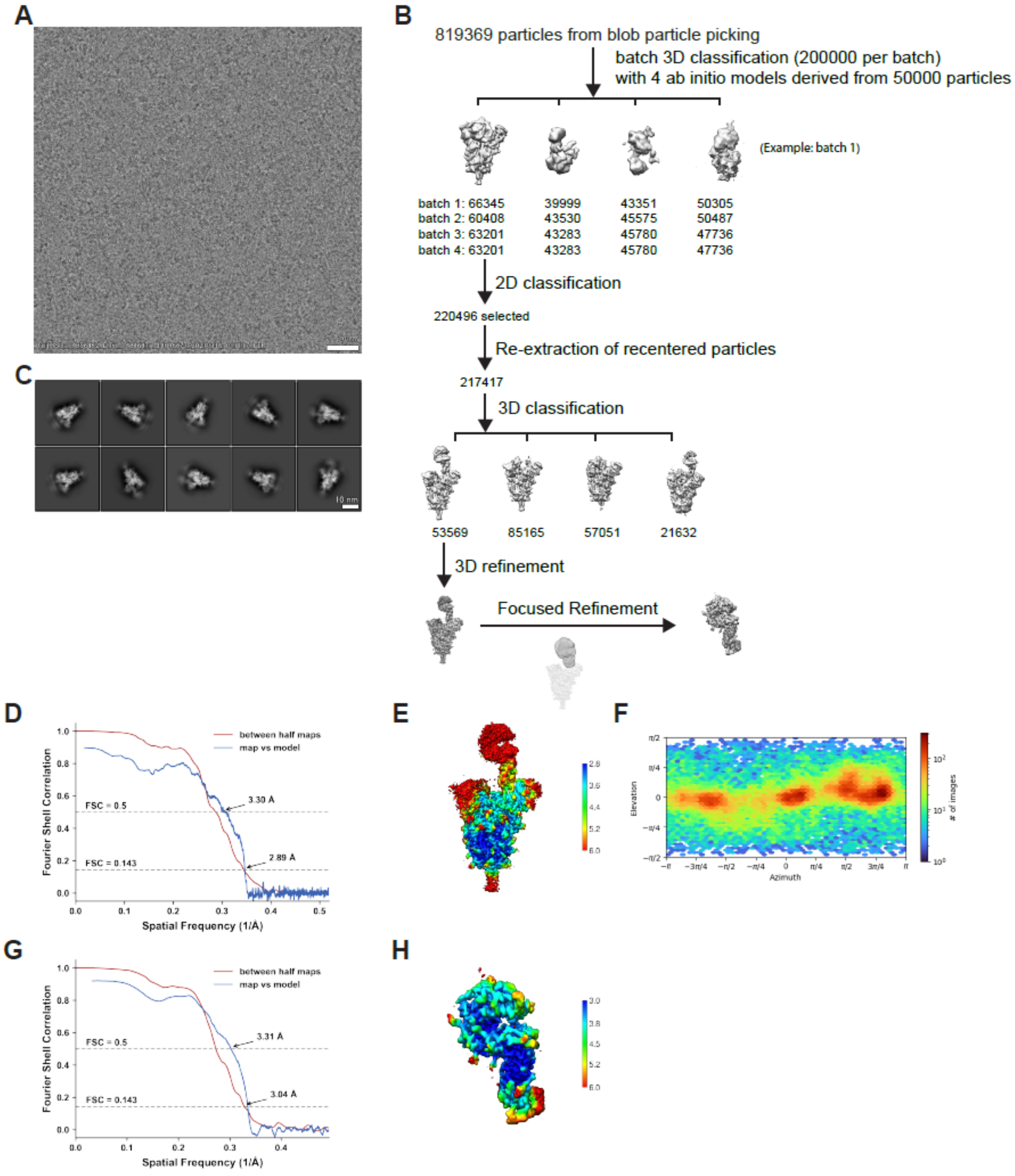

Supplemental Fig. 19. Cryo-EM data processing and validation for complex of Beta spike protein ectodomain and human ACE2. (A) Representative cryo-EM micrograph. **(B)** Workflow of cryo-EM image processing. **(C)** Representative 2D classes. **(D-F)** FSC curves (D), local resolution (E) and viewing direction distribution plot (F) of global refinement. **(G-H)** FSC curves (G) and local resolution (H) of focused refinement.

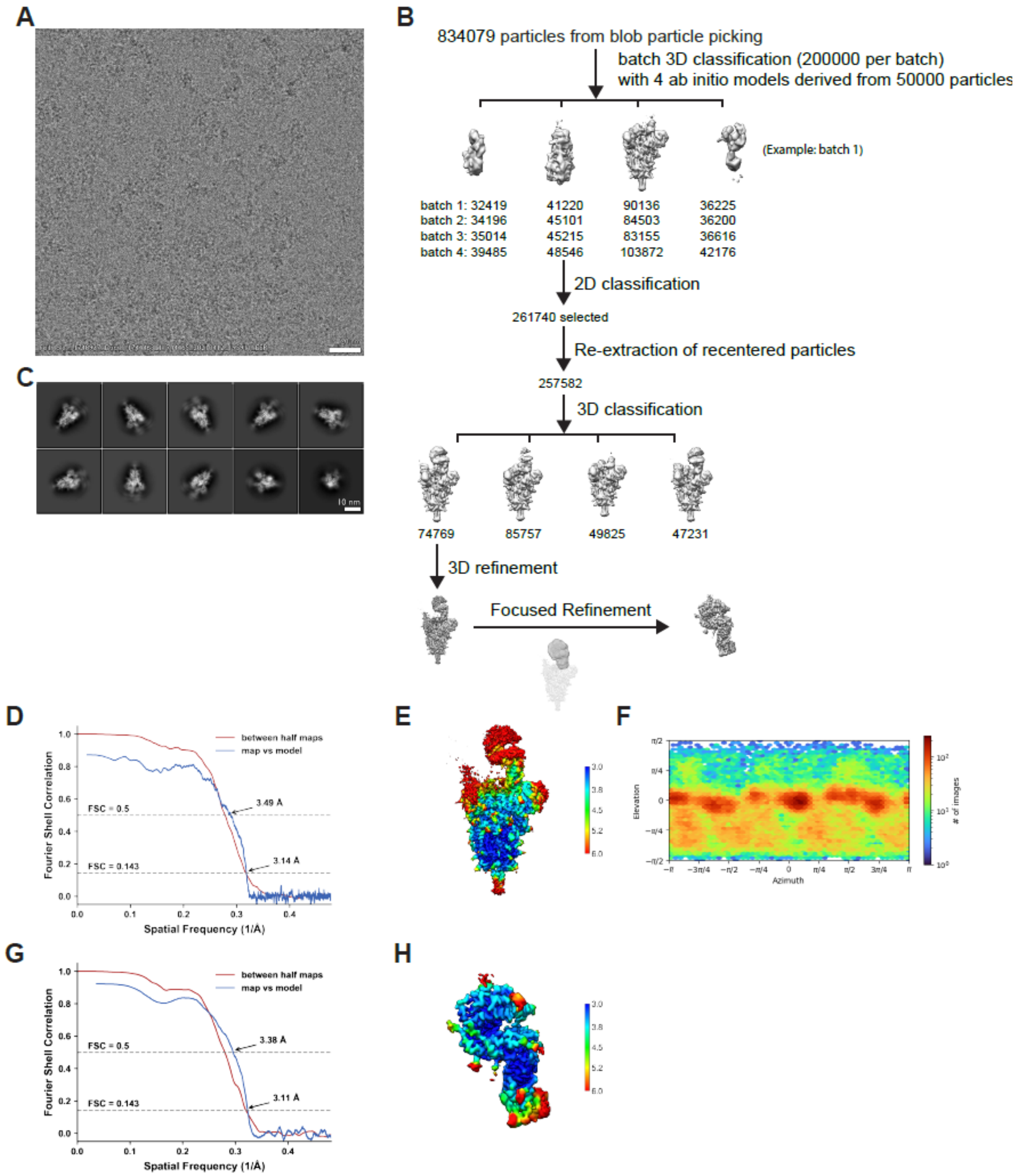

Supplemental Fig. 20. Cryo-EM data processing and validation for complex of Epsilon spike protein ectodomain and human ACE2. (A) Representative cryo-EM micrograph. **(B)** Workflow of cryo-EM image processing. **(C)** Representative 2D classes. **(D-F)** FSC curves (D), local resolution (E) and viewing direction distribution plot (F) of global refinement. **(G-H)** FSC curves (G) and local resolution (H) of focused refinement.

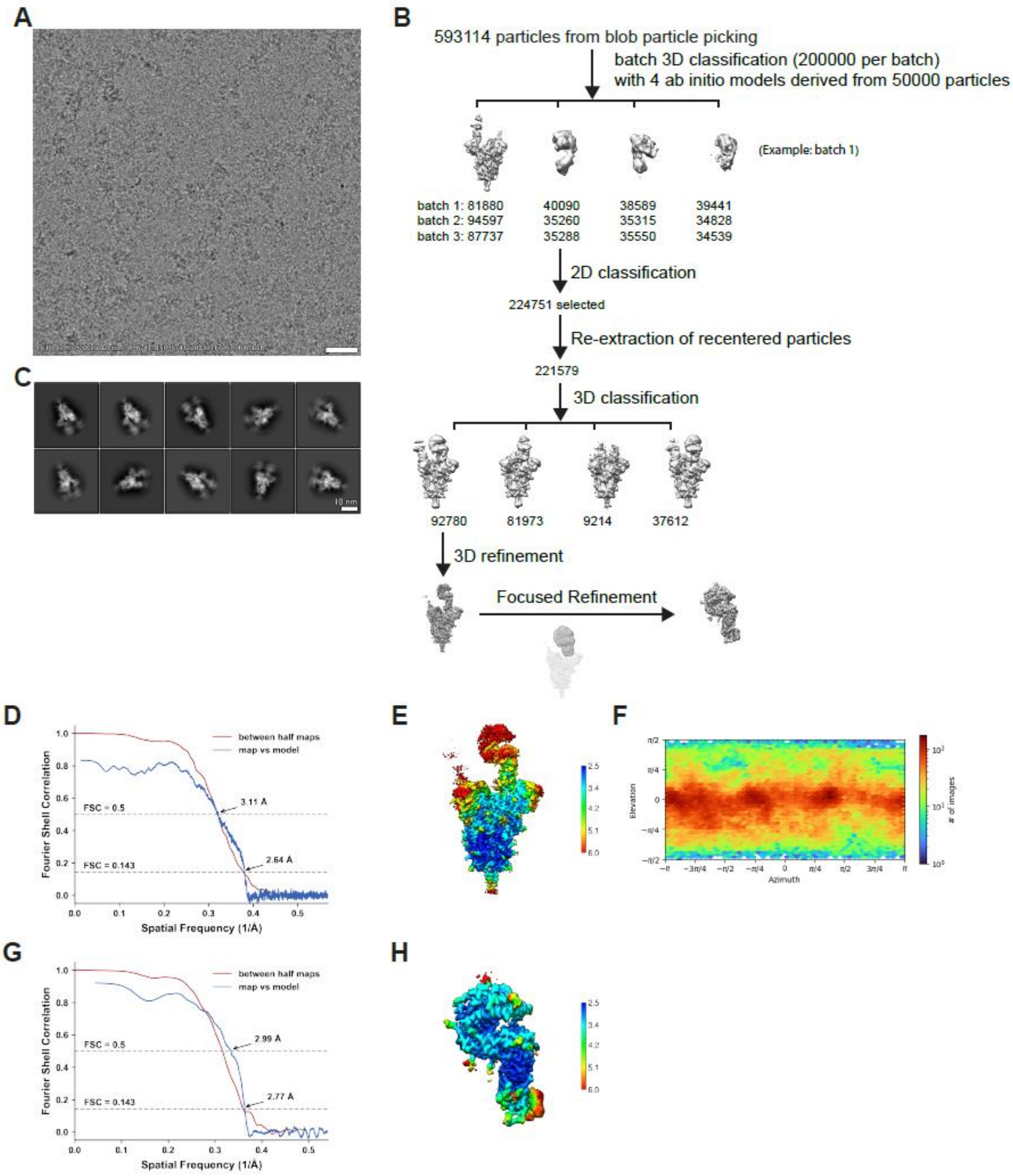

Supplemental Fig. 21. Cryo-EM data processing and validation for complex of Gamma spike protein ectodomain and human ACE2. (A) Representative cryo-EM micrograph. **(B)** Workflow of cryo-EM image processing. **(C)** Representative 2D classes. **(D-F)** FSC curves (D), local resolution (E) and viewing direction distribution plot (F) of global refinement. **(G-H)** FSC curves (G) and local resolution (H) of focused refinement.

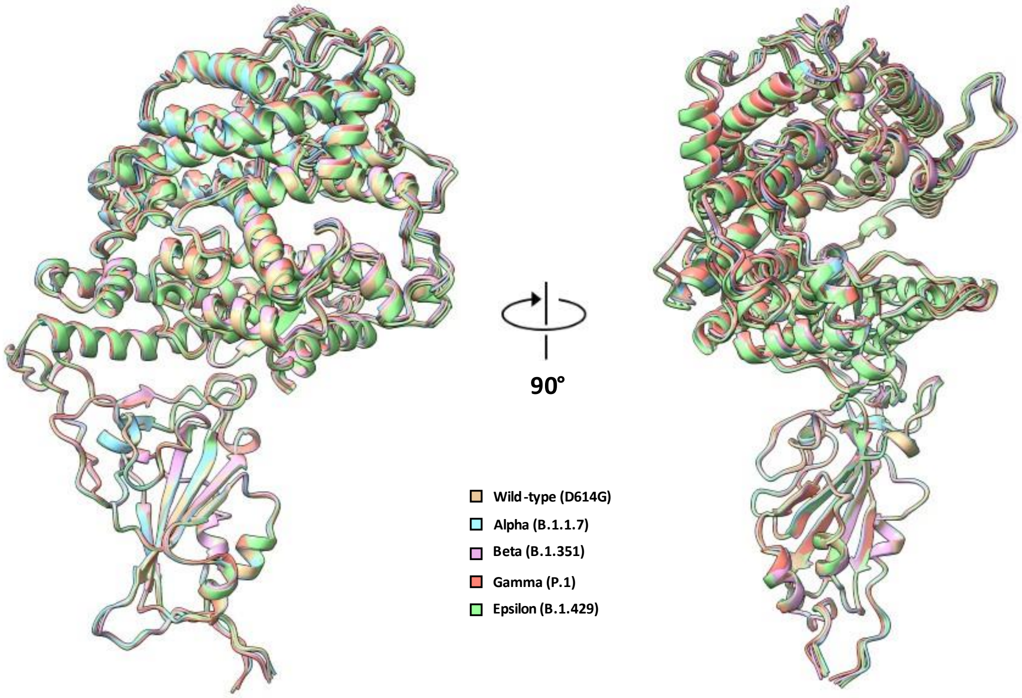

Supplemental Fig. 22. Superposition of RBD-ACE2 local models. Models were aligned using the RBD for the superposition. The following PDB was used for the wild-type spike RBD-ACE2 model: 7SXY.

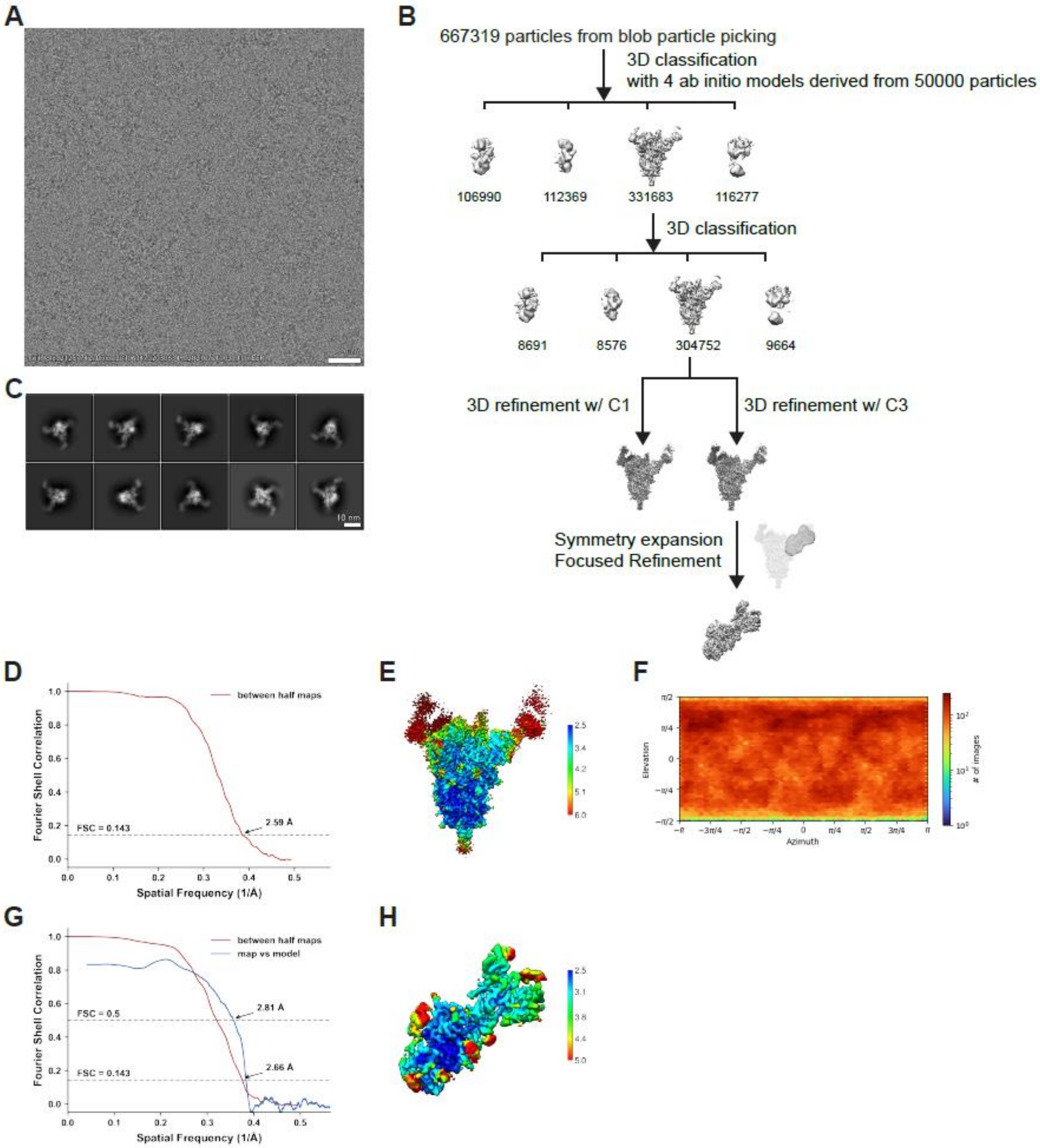

Supplemental Fig. 23. Cryo-EM data processing and validation for complex of Gamma spike protein ectodomain and 4-A8 Fab. (A) Representative cryo-EM micrograph. **(B)** Workflow of cryo-EM image processing. **(C)** Representative 2D classes. **(D-F)** FSC curves **(D)**, local resolution **(E)** and viewing direction distribution plot **(F)** of global refinement. **(G-H)** FSC curves **(G)** and local resolution **(H)** of focused refinement.

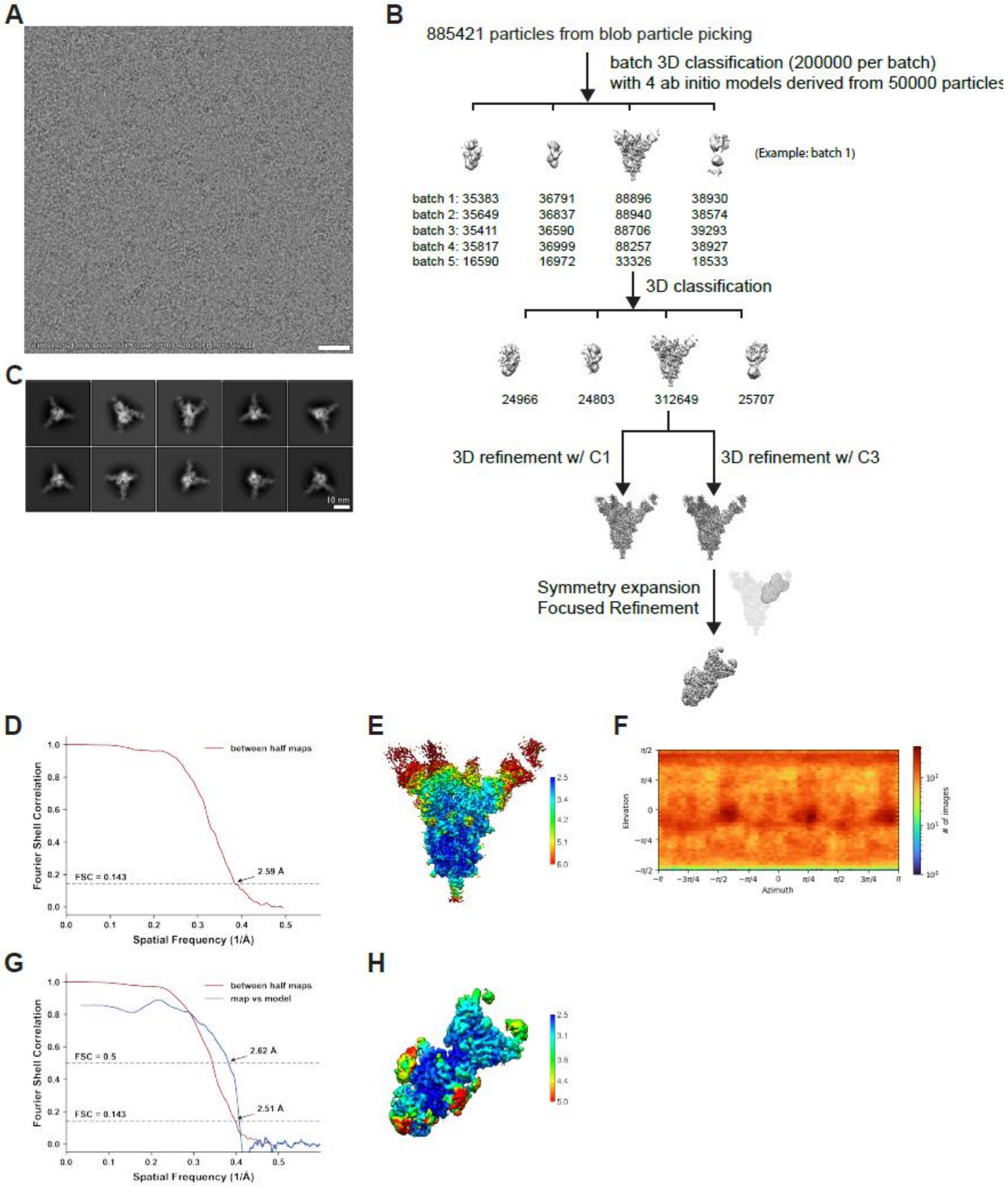

Supplemental Fig. 24. Cryo-EM data processing and validation for complex of Gamma spike protein ectodomain and 4-8 Fab. (A) Representative cryo-EM micrograph. **(B)** Workflow of cryo-EM image processing. **(C)** Representative 2D classes. **(D-F)** FSC curves **(D)**, local resolution **(E)** and viewing direction distribution plot **(F)** of global refinement. **(G-H)** FSC curves **(G)** and local resolution **(H)** of focused refinement.

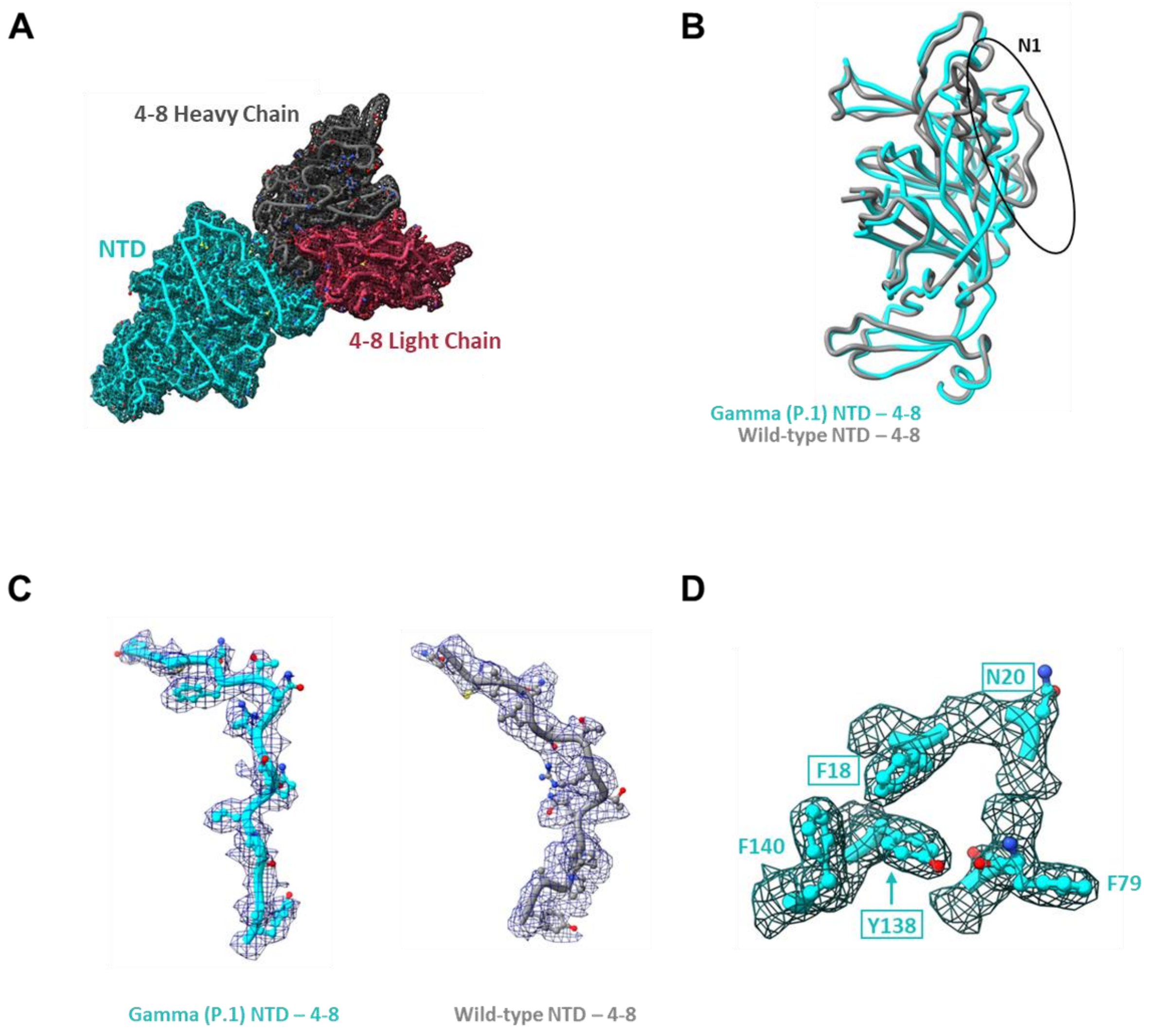

Supplemental Fig. 25. Structure of the Gamma variant NTD bound by 4-8 reveals rearrangement of the N1 loop. **(A)** Local cryoEM density map and model of the Gamma variant S protein bound to 4-8 at 2.51 Å **(B)** Superposition of 4-8-bound Gamma and wild-type NTD models showing N1 loop rearrangement. **(C)** Density and models for the N1 loops compared in panel (B). **(D)** Positioning of the L18->F, D138->Y, and T20->N mutations and adjacent residues in the Gamma NTD. **(D)** Superposition of residues shown in (C) with WT residues demonstrates steric incompatibilities. Areas of steric clashes are indicated by dashed ovals. Mutated residues are indicated as boxed labels. The wild-type – 4-8 model (PDB: 7LQV) was used for superpositions and is shown in grey throughout the figure.

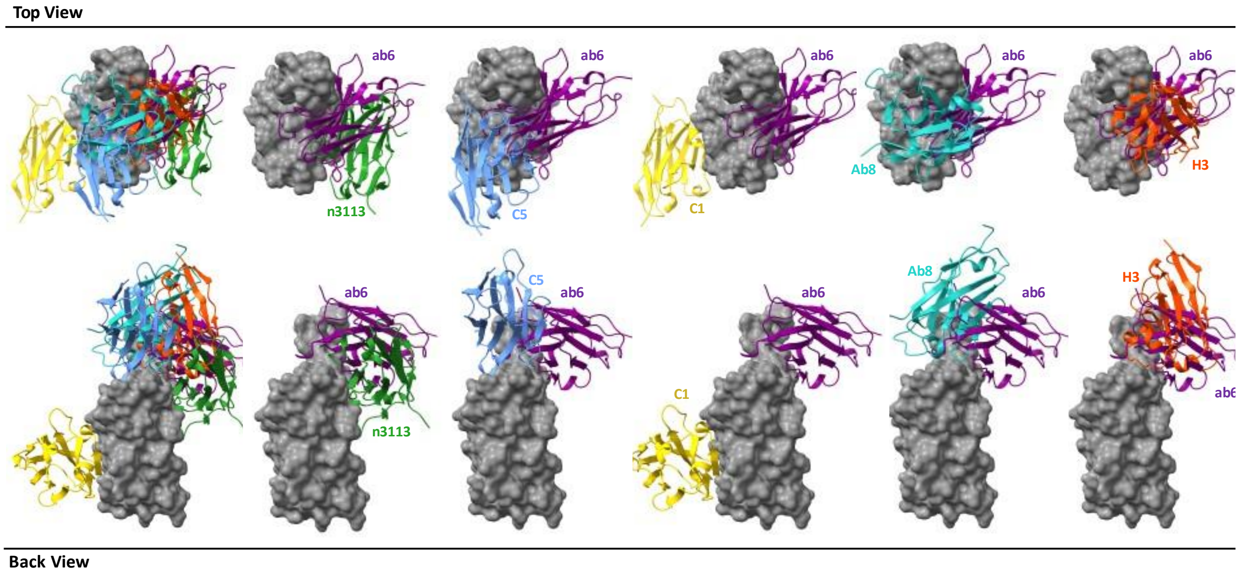

Supplemental Fig. 26. Footprint comparison between ab6 and selected RBD-directed V_H domains. The RBD is depicted as a grey molecular surface and antibodies are depicted as colourized cartoon models. The following PDB files were utilized: 7VNB (n3113), 7OAP (H3 and C1), 7MJI (ab8), 7OAO (C5). The RBD model from the ab6-RBD complex is shown for all antibody complexes for ease of visualization. For superpositions, structures were aligned using the RBD.

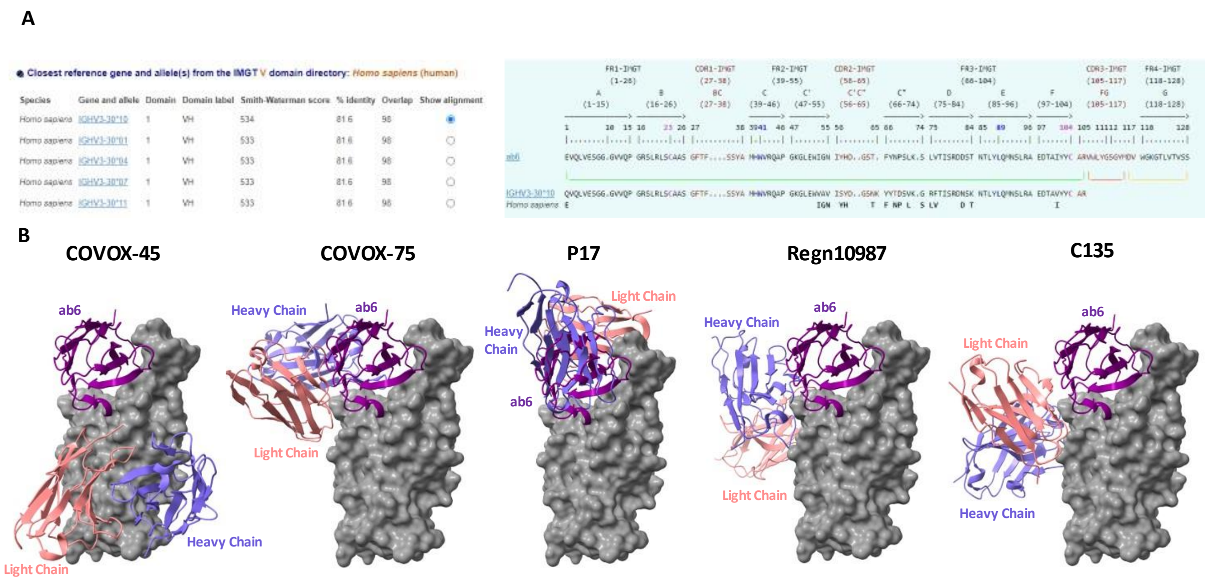

Supplemental Fig. 27. Sequence analysis of ab6 and footprint comparison between ab6 and selected RBD-directed IGVH3-30 encoded antibodies. (A) Closest reference IMGT V domains to the ab6 amino acid sequence (left) and alignment of the ab6 amino acid sequence with the IGHV3-30-10 sequence (right). (**B)** Footprint comparison between ab6 and selected IGHV3-30 antibodies. The RBD is depicted as a grey molecular surface and antibodies are depicted as colourized cartoon models. The following PDB files were utilized: 7BEI (COVOX-45), 7BEN (COVOX-75), 7CWO (P17) 6XDG (Regn10987), 7K8Z (C135). The RBD model from the ab6-RBD complex is shown for all antibody complexes for ease of visualization. For superpositions, structures were aligned using the RBD.

Supplemental Table 1: CryoEM data collection, processing, refinement, and validation parameters for the structures reported in this publication.

Structure:	Alpha	Alpha + ACE2		Beta	Beta + ACE2		Gamma	Gamma + ACE2		Gamma + 4-8	
		global refinement	focus refinement		global refinement	focus refinement		global refinement	focus refinement	global refinement	focus refinement
	EMD-27502	EMD-27503	EMD-27504	EMD-27505	EMD-27506	EMD-27507	EMD-27508	EMD-27509	EMD-27510	EMD-27511	EMD-27512
	PDB 8DLI	PDB 8DLJ	PDB 8DLK	PDB 8DLL	PDB 8DLM	PDB 8DLN	PDB 8DLO	PDB 8DLP	PDB 8DLQ		8DLR
**Data collection**											
Microscope	Titan Krios G4	Titan Krios G4		Titan Krios G4	Titan Krios G4		Titan Krios G4	Titan Krios G4		Titan Krios G4	
Detector	Falcon4	Falcon4		Falcon4	Falcon4		Falcon4	Falcon4		Falcon4	
Voltage (kV)	300	300		300	300		300	300		300	
Nominal magnification	155,000	155,000		155,000	155,000		155,000	155,000		155,000	
Defocus range (μm)	-3.0 to -0.5	-3.0 to -0.5		-3.0 to -0.5	-3.0 to -0.5		-3.0 to -0.5	-3.0 to -0.5		-3.0 to -0.5	
Physical pixel (Å)	0.5	0.5		0.5	0.5		0.5	0.5		0.5	
Electron dose ($e^-/Å^2$)	40	40		40	40		40	40		40	
Exposure rate ($e^-/Å^2$/sec)	24	24		24	24		24	24		24	
Format of movies	EER	EER		EER	EER		EER	EER		EER	
Number of raw frames	399	399		399	399		399	399		399	
Number of movies	4,050	7,452		2,997	10,667		4,410	9,720		9,709	
**Data processing**											
Number of fractions	40	40		40	40		40	40		40	
Number of extracted particles	292,930	551,927		215,447	819,369		405,128	593,114		885,421	
Number of particles for final map	104,788	50,298		94,503	53,569		194,867	92,780		312,649	937,947*
Symmetry imposed	C1	C1	C1	C1	C1	C1	C1	C1	C1	C1	C1
Resolution (Å)	2.56	2.91	3.04	2.56	2.89	3.04	2.25	2.64	2.77	2.59	2.51
FSC threshold	0.143	0.143	0.143	0.143	0.143	0.143	0.143	0.143	0.143	0.143	0.143
**Refinement**											
Initial model used	7MJG	7MJM,7MJN	7MJN	7MJG	7MJM,7MJN	7MJN	7MJG	7MJM,7MJN	7MJN	/	7LXY
Map sharpening B-factor ($Å^2$)	59.3	59.1	90.1	55.8	58.7	88.4	34.7	57.6	81.4	53.7	65.9
Composition (#)											
Atoms	25,154	28,498	6,554	25,095	26,852	6,553	25,257	40,095	6,552	/	4,027
Residues	3,110	3,508	797	3,102	3,303	797	3,120	4,908	797	/	505
Ligands	NAG:60	NAG:64	NAG:7	NAG:60	NAG:62	NAG:7	NAG:60	NAG:78	NAG:7	/	NAG:6
B-factor ($Å^2$)											
Protein (min/max/mean)	53.14/303.75/131.70	60.02/446.03/167.98	85.86/229.48/128.92	52.70/678.37/160.77	58.66/528.32/200.62	77.04/227.70/120.96	34.04/225.84/111.10	21.36/369.82/196.05	63.90/195.10/101.53	/	49.52/112.03/78.68
Ligand (min/max/mean)	80.69/249.33/139.50	87.77/359.02/163.13	135.50/162.85/148.61	74.51/511.64/147.42	81.84/526.84/176.24	122.80/145.68/132.04	52.35/221.80/114.85	52.35/419.30/185.86	107.50/130.18/117.54	/	77.59/106.81/89.57
Bonds (RMSD)											
Length (Å) (# > 4σ)	0.004 (0)	0.004 (0)	0.005 (0)	0.004 (0)	0.004 (0)	0.005 (0)	0.005 (0)	0.004 (0)	0.006 (0)	/	0.006 (0)
Angles (°) (# > 4σ)	0.794 (6)	0.817 (8)	0.847 (2)	0.746 (6)	0.762 (8)	0.845 (3)	0.746 (4)	0.765 (12)	0.896 (3)	/	0.789 (1)
CC_mask	0.81	0.82	0.85	0.82	0.82	0.85	0.82	0.75	0.87	/	0.84
**Validation**											
Ramachandran plot											
Residues favored (%)	98.04	98.26	98.11	97.18	98.21	98.23	97.40	97.80	98.23	/	97.37
Residues disallowed (%)	0.00	0.03	0.00	0.00	0.00	0.00	0.00	0.04	0.00	/	0.00
Rotamer outliers (%)	0.07	0.13	0.29	0.00	0.03	0.29	0.00	0.12	0.43	/	0.00
Clash score	3.61	3.45	2.58	3.13	3.33	2.74	3.05	2.91	2.97	/	3.79
MolProbity score	1.15	1.14	1.04	1.25	1.12	1.06	1.21	1.13	1.09	/	1.29

* Derived by symmetry expansion of particles from global refinement with C3 symmetry.

Table S1 continued

Gamma + 4A8		Epsilon	Epsilon + ACE2		Epsilon + S2M11	Epsilon + VH Ab6		S(D614G) + VH Ab6	
global refinement	focus refinement		global refinement	focus refinement		global refinement	focus refinement	global refinement	focus refinement
EMD-27513	EMD-27514	EMD-27515	EMD-27516	EMD-27517	EMD-27518	EMD-27519	EMD-27520	EMD-27521	EMD-27522
	PDB 8DLS	PDB 8DLT	PDB 8DLU	PDB 8DLV	PDB 8DLW	PDB 8DLX	PDB 8DLY	PDB 8DLZ	PDB 8DM0
Titan Krios G4		Titan Krios G4	Titan Krios G4		Titan Krios G4	Titan Krios G4		Titan Krios G4	
Falcon4		Falcon4	Falcon4		Falcon4	Falcon4		Falcon4	
300		300	300		300	300		300	
155,000		155,000	155,000		155,000	155,000		155,000	
-3.0 to -0.5		-3.0 to -0.5	-3.0 to -0.5		-3.0 to -0.5	Falcon4		Falcon4	
0.5		0.5	0.5		0.5	0.5		0.5	
40		40	40		40	40		40	
24		24	24		24	24		24	
EER		EER	EER		EER	EER		EER	
399		399	399		399	399		399	
8,507		4,482	10,692		7,686	10,017		8,857	
40		40	40		40	40		40	
667,319		359,342	834,079		533,458	730,932		693,104	
304,752	914,256*	157,853	74,769		124,473	122,910		121,117	
C1	C1	C1	C1	C1	C3	C1	C1	C1	C1
2.59	2.66	2.40	3.14	3.11	2.16	2.45	3.00	2.57	3.21
0.143	0.143	0.143	0.143	0.143	0.143	0.143	0.143	0.143	0.143
/	7LXY	7MJG	7MJM,7MJN	7MJN	7K43	7MJG, 7MJI	7MJI	7MJG, 7MJI	7MJI
52.3	75.1	58.6	72.1	94.2	55.8	56.3	77.0	42.5	74.0
/	4,017	25,245	35,137	6,553	30,267	24,293	2,483	22,974	2,480
/	495	3,120	4,312	797	3,768	3,005	314	2,842	314
/	NAG:7	NAG:60	NAG:72	NAG:7	BMA:3; NAG:72; FUC:3	NAG:59	NAG:1	NAG:57	NAG:1
/	50.02/133.68/96.97	53.70/382.99/132.79	56.65/575.12/250.82	83.10/212.32/114.94	45.58/191.33/89.64	57.17/371.39/132.29	71.28/144.28/105.61	51.22/285.85/118.30	55.77/132.67/98.26
/	77.67/131.36/99.80	73.64/298.82/134.51	88.85/588.23/225.19	121.24/146.32/130.07	79.27/202.53/115.88	81.36/307.72/135.41	105.34/105.34/105.34	71.35/231.91/124.66	84.59/84.59/84.59
/	0.006 (0)	0.004 (0)	0.004 (0)	0.005 (0)	0.006 (0)	0.004 (0)	0.005 (0)	0.004 (0)	0.006 (0)
/	0.904 (3)	0.728 (6)	0.770 (9)	0.871 (2)	1.021 (22)	0.797 (7)	0.934 (0)	0.794 (7)	1.001 (4)
/	0.83	0.84	0.79	0.85	0.86	0.84	0.81	0.83	0.80
/	95.24	98.27	98.22	98.49	97.65	97.83	96.45	97.99	96.45
/	0.00	0.00	0.00	0.00	0.05	0.00	0.00	0.00	0.00
/	0.00	0.00	0.08	0.29	0.19	0.08	0.37	0.12	0.00
/	4.45	2.75	2.92	2.58	1.95	3.50	1.86	3.31	0.83
/	1.55	1.06	1.08	1.04	1.04	1.18	1.18	1.12	0.99

Supplemental Materials References

1. Saville, J. W. *et al.* Structural and biochemical rationale for enhanced spike protein fitness in delta and kappa SARS-CoV-2 variants. *Nat. Commun.* **13**, 742 (2022).
