# Supplementary material for: SARS-CoV-2 variants of concern: spike protein mutational analysis and epitope for broad neutralization
Source: Nat Commun. 2022 Aug 18;13:4696. doi: 10.1038/s41467-022-32262-8 (PMC9388680; doi:10.1038/s41467-022-32262-8)
Supplement: Supplementary file 2 — Reporting Summary [file 41467_2022_32262_MOESM2_ESM.pdf]

nature portfolio

Corresponding author(s): Dr. Sriram Subramaniam

Last updated by author(s): Jun 24, 2022

Reporting Summary

Nature Portfolio wishes to improve the reproducibility of the work that we publish. This form provides structure for consistency and transparency in reporting. For further information on Nature Portfolio policies, see our Editorial Policies and the Editorial Policy Checklist.

Statistics

For all statistical analyses, confirm that the following items are present in the figure legend, table legend, main text, or Methods section.

n/a	Confirmed	
☐	☒	The exact sample size (*n*) for each experimental group/condition, given as a discrete number and unit of measurement
☐	☒	A statement on whether measurements were taken from distinct samples or whether the same sample was measured repeatedly
☐	☒	The statistical test(s) used AND whether they are one- or two-sided<br>*Only common tests should be described solely by name; describe more complex techniques in the Methods section.*
☒	☐	A description of all covariates tested
☒	☐	A description of any assumptions or corrections, such as tests of normality and adjustment for multiple comparisons
☐	☒	A full description of the statistical parameters including central tendency (e.g. means) or other basic estimates (e.g. regression coefficient) AND variation (e.g. standard deviation) or associated estimates of uncertainty (e.g. confidence intervals)
☐	☒	For null hypothesis testing, the test statistic (e.g. *F*, *t*, *r*) with confidence intervals, effect sizes, degrees of freedom and *P* value noted<br>*Give P values as exact values whenever suitable.*
☒	☐	For Bayesian analysis, information on the choice of priors and Markov chain Monte Carlo settings
☒	☐	For hierarchical and complex designs, identification of the appropriate level for tests and full reporting of outcomes
☒	☐	Estimates of effect sizes (e.g. Cohen's *d*, Pearson's *r*), indicating how they were calculated

Our web collection on statistics for biologists contains articles on many of the points above.

Software and code

Policy information about availability of computer code

Data collection	EPU 2 automated acquisition (Thermo Fisher Scientific; https://www.thermofisher.com/us/en/home/electron-microscopy/products/software-em-3d-vis/epu-software.html)

Data analysis	GraphPad Prism (GraphPad 9.0; https://www.graphpad.com/scientific-software/prism/)<br><br>UCSF Chimera (v.1.16) (Eric F. Pettersen TDG, Conrad C. Huang, Gregory S. Couch, Daniel M. Greenblatt, Elaine C. Meng, Thomas E. Ferrin. UCSF Chimera—A visualization system for exploratory research and analysis. Journal of Computational Chemistry 25, 1605-1612 (2004). https://www.cgl.ucsf.edu/chimera/)<br><br>RELION 3.1 (Scheres SHW. RELION: Implementation of a Bayesian approach to cryo-EM structure determination. Journal of Structural Biology 180, 519-530 (2012). https://github.com/3dem/relion/releases/tag/3.1.0)<br><br>crYOLO (version 1.7.4) (Wagner T, et al. SPHIRE-crYOLO is a fast and accurate fully automated particle picker for cryo-EM. Communications Biology 2, (2019). https://pypi.org/project/cryolo/)<br><br>cryoSPARC live (v3.0.1) (Punjani A, Rubinstein JL, Fleet DJ, Brubaker MA. cryoSPARC: algorithms for rapid unsupervised cryo-EM structure determination. Nature Methods 14, 290-296 (2017). https://cryosparc.com/live)<br><br>MolProbity (v4.5.1) (Chen, V. B. et al. MolProbity: all-atom structure validation for macromolecular crystallography. Acta Crystallogr. D. Biol. Crystallogr. 66, 12–21 (2010). http://molprobity.biochem.duke.edu/)<br><br>FlowJo (v10.8.1) (Becton, Dickinson and Company. https://www.flowjo.com/)

For manuscripts utilizing custom algorithms or software that are central to the research but not yet described in published literature, software must be made available to editors and reviewers. We strongly encourage code deposition in a community repository (e.g. GitHub). See the Nature Portfolio guidelines for submitting code & software for further information.

Data

Policy information about availability of data

All manuscripts must include a data availability statement. This statement should provide the following information, where applicable:

- Accession codes, unique identifiers, or web links for publicly available datasets
- A description of any restrictions on data availability
- For clinical datasets or third party data, please ensure that the statement adheres to our policy

The atomic models and cryo-EM density maps have been deposited into the Protein Data Bank (PDB) and Electron Microscopy Data Bank (EMDB) as follows: Alpha (B.1.1.7) apo spike protein: PDB 8DLI and EMD-27502, Alpha (B.1.1.7) spike protein-ACE2 complex (global): PDB 8DLJ and EMD-27503, Alpha (B.1.1.7) spike protein-ACE2 complex (focused): PDB 8DLK and EMD-27504, Beta (B.1.351) apo spike protein: PDB 8DLL and EMD-27505, Beta (B.1.351) spike protein-ACE2 complex (global): PDB 8DLM and EMD-27506, Beta (B.1.351) spike protein-ACE2 complex (focused): PDB 8DLN and EMD-27507, Gamma (P.1) apo spike protein: PDB 8DLO and EMD-27508, Gamma (P.1) spike protein-ACE2 complex (global): PDB 8DLP and EMD-27509, Gamma (P.1) spike protein-ACE2 complex (local): PDB 8DLQ and EMD-27510, Gamma (P.1) spike protein-4-8 complex (global): EMD-27511, Gamma (P.1) spike protein-4-8 complex (focused): PDB 8DLR and EMD-27512, Gamma (P.1) spike protein-4A8 complex (global): EMD-27513, Gamma (P.1) spike protein-4A8 complex (focused): PDB 8DLS and EMD-27514,Epsilon (B.1.429) apo spike protein: PDB 8DLT and EMD-27515, Epsilon (B.1.429) spike protein-ACE2 complex (global): PDB 8DLU and EMD-27516, Epsilon (B.1.429) spike protein-ACE2 complex (focused): PDB 8DLV and EMD-27517, Epsilon (B.1.429) spike protein-S2M11 complex: PDB 8DLW and EMD-2751, Epsilon (B.1.429) spike protein-VH ab6 complex (global): PDB 8DLX and EMD-27519, , Epsilon (B.1.429) spike protein-VH ab6 complex (focused): PDB 8DLY and EMD-27520, D614G spike protein-VH ab6 complex (global): PDB 8DLZ and EMD-27521, D614G spike protein-VH ab6 complex (focused): PDB 8DM0 and EMD-27522.

Field-specific reporting

Please select the one below that is the best fit for your research. If you are not sure, read the appropriate sections before making your selection.

- [x] Life sciences
- [ ] Behavioural & social sciences
- [ ] Ecological, evolutionary & environmental sciences

For a reference copy of the document with all sections, see nature.com/documents/nr-reporting-summary-flat.pdf

Life sciences study design

All studies must disclose on these points even when the disclosure is negative.

Sample size	Sample size was not predetermined and the sample size presented in this manuscript reflects the availability of vaccine-induced or convalescent patient-derived serum samples.
Data exclusions	No data was excluded from this study.
Replication	For all biochemical assays- multiple complimentary types of experiments were used to test the hypotheses presented in this study.
Randomization	Our samples were not randomized as all samples were treated identically within the same experimental design.
Blinding	Blinding was not relevant to this study as all measurements were obtained by quantitative biochemical assays and not through subjective means.

Reporting for specific materials, systems and methods

We require information from authors about some types of materials, experimental systems and methods used in many studies. Here, indicate whether each material, system or method listed is relevant to your study. If you are not sure if a list item applies to your research, read the appropriate section before selecting a response.

Materials & experimental systems

n/a	Involved in the study	
☐	☒	Antibodies
☐	☒	Eukaryotic cell lines
☒	☐	Palaeontology and archaeology
☒	☐	Animals and other organisms
☐	☒	Human research participants
☒	☐	Clinical data
☒	☐	Dual use research of concern

Methods

n/a	Involved in the study	
☒	☐	ChIP-seq
☒	☐	Flow cytometry
☒	☐	MRI-based neuroimaging

Antibodies

Antibodies used

VH ab8 (Li W, et al. PNAS 2020; Li W, et al. Cell 2020)
IgG1 ab1 (Li W, et al. PNAS 2020; Li W, et al. Cell 2020)
Fab S309 (Pinto D, et al. Nature 2020)
Fab S2M11 (Tortorici MA, et al. Science 2020)
Fab 4A8 (Chi X, et al. Science 2020)
Fab 4-8 (Liu L, et al. Nature 2020)
Goat anti-human IgG - HRP (Jackson ImmunoResearch, Cat. # 109-035-088)
Anti-SARS-CoV-2 spike glycoprotein antibody - Coronavirus (ab272504)
IgG (H+L) Cross-Adsorbed Goat anti-Rabbit, DyLight 680, (Invitrogen, Cat. # PI35569
Goat anti-Mouse IgG (H+L) Cross-Adsorbed Secondary Antibody, Alexa Fluor 647, (Invitrogen, Cat. # A-21235)
Anti-Strep-Tag Antibody (BIO-RAD, Cat. # MCA2489)

Validation

The validation of each primary antibody is described in their associated publication, as referenced in the above section.
Validation documentation for the Goat anti-human IgG - HRP antibody can be found at this webpage: https://www.jacksonimmuno.com/catalog/products/109-035-088, for the anti-SARS-CoV-2 spike glycoprotein antibody at this webpage: https://www.abcam.com/sars-cov-2-spike-glycoprotein-antibody-coronavirus-ab272504.html#description_images_1 , IgG (H+L) Cross-Adsorbed Goat anti-Rabbit: https://www.fishersci.com/shop/products/goat-anti-rabbit-igg-h-l-dylight-680-polyclonal-thermo-scientific-pierce-dylight-680/PI35569, for the Goat anti-Mouse IgG (H+L) Cross-Adsorbed Secondary Antibody, Alexa Fluor 647: https://www.thermofisher.com/antibody/product/Goat-anti-Mouse-IgG-H-L-Cross-Adsorbed-Secondary-Antibody-Polyclonal/A-21235 and for Anti-Strep-Tag Antibody: https://www.bio-rad-antibodies.com/monoclonal/synthetic-peptide-strep-tag-classic-antibody-strep-tag-ii-mca2489.html?f=purified

Eukaryotic cell lines

Policy information about cell lines

Cell line source(s)

Expi293F (Thermo Fisher Scientific; cat# A14527)
HEK293T-ACE2-TMPRSS2 cells (BEI Resources; cat# NR-55293)
Vero E6 cells (ATCC; cat# CRL-1586)

Authentication

Expi293F - please see this webpage for authentication documentation: https://www.thermofisher.com/order/catalog/product/A14527#/A14527

HEK293T-ACE2-TMPRSS2 cells - please see this webpage for authentication documentation: https://www.beiresources.org/Catalog/cellBanks/NR-55293.aspx

Vero E6 cells - please see this webpage for authentication documentation: https://www.atcc.org/products/crl-1586

Mycoplasma contamination

Cell lines were not tested for mycoplasma contamination, as they were used solely for protein expression and pseudovirus neutralization assays.

Commonly misidentified lines
(See ICLAC register)

No commonly misidentified cell lines were used in this study.

Human research participants

Policy information about studies involving human research participants

Population characteristics	Biospecimen type: Blood serum<br>Anatomical or collection site: Venipuncture blood draw<br>Biospecimen disease status: +/- confirmed COVID-19 diagnosis who have fully recovered from infection and +/- COVID19 vaccination.<br>Clinical characteristics of patients: Age, sex<br>Vital state: Alive<br>Diagnosis: +/- confirmed COVID-19 diagnosis who have fully recovered from infection and +/- COVID19 vaccination.<br>Collection mechanism and parameters: Venipuncture blood draw.<br>Biospecimen storage: Patient-derived serum samples were stored at -80C until use. Maximum storage duration: 1 year.<br>Composition assessment and selection: All provided samples were included in this study.
Recruitment	Recruitment was performed as outlined here: http://www.bccdc.ca/health-professionals/clinical-resources/covid-19-care/covid-19-serology-care-covid-study
Ethics oversight	Patient derived sera samples were collected according to the CARE COVID Study (http://www.bccdc.ca/health-professionals/clinical-resources/covid-19-care/covid-19-serology-care-covid-study) with ethics approval from the UBC Clinical Research Ethics Board.

Note that full information on the approval of the study protocol must also be provided in the manuscript.
